# Replication of Simulated Prebiotic Amphiphilic Vesicles in a Finite Environment Exhibits Complex Behavior That Includes High Progeny Variability and Competition

**DOI:** 10.1089/ast.2016.1615

**Published:** 2018-04-01

**Authors:** Don L. Armstrong, Doron Lancet, Raphael Zidovetzki

**Affiliations:** ^1^ Institute for Genomic Biology, University of Illinois at Urbana-Champaign, Urbana, Illinois, USA.; ^2^ Department of Cell Biology and Neuroscience, University of California, Riverside, California, USA.; ^3^ Department of Molecular Genetics, Weizmann Institute of Science, Rehovot, Israel.

## Abstract

We studied the simulated replication and growth of prebiotic vesicles composed of 140 phospholipids and cholesterol using our R-GARD (Real Graded Autocatalysis Replication Domain) formalism that utilizes currently extant lipids that have known rate constants of lipid-vesicle interactions from published experimental data. R-GARD normally modifies kinetic parameters of lipid-vesicle interactions based on vesicle composition and properties. Our original R-GARD model tracked the growth and division of one vesicle at a time in an environment with unlimited lipids at a constant concentration. We explore here a modified model where vesicles compete for a finite supply of lipids. We observed that vesicles exhibit complex behavior including initial fast unrestricted growth, followed by intervesicle competition for diminishing resources, then a second growth burst driven by better-adapted vesicles, and ending with a final steady state. Furthermore, in simulations without kinetic parameter modifications (“invariant kinetics”), the initial replication was an order of magnitude slower, and vesicles' composition variability at the final steady state was much lower. The complex kinetic behavior was not observed either in the previously published R-GARD simulations or in additional simulations presented here with only one lipid component. This demonstrates that both a finite environment (inducing selection) and multiple components (providing variation for selection to act upon) are crucial for portraying evolution-like behavior. Such properties can improve survival in a changing environment by increasing the ability of early protocellular entities to respond to rapid environmental fluctuations likely present during abiogenesis both on Earth and possibly on other planets. This *in silico* simulation predicts that a relatively simple *in vitro* chemical system containing only lipid molecules might exhibit properties that are relevant to prebiotic processes. Key Words: Phospholipid vesicles—Prebiotic compartments—Prebiotic vesicle competition—Prebiotic vesicle variability. Astrobiology 18, 419–430.

## 1. Introduction

One of the first questions of early evolution is when a specific form of information transfer emerged: “What came first?” The “RNA World” hypothesis suggests that the presence of informational biopolymers such as RNA was an absolute requirement for information transfer and life's emergence. RNA is hypothesized to be the information molecule because RNA not only has the capacity of storing and replicating sequence-based (genetic or linear) information by a templating mechanism but also can catalyze reactions (Joyce, 2002; Copley *et al.,* 2007). It has been further suggested that, after the emergence of RNA-based biopolymers, RNA was sequestered into compartments, isolating RNA from the environment, thereby protecting the informational molecules, and potentially leading to cellular life (Joyce, 2002; Copley *et al.,* 2007).

An alternative, “Lipid World” hypothesis (Segré *et al.,* 2001a), suggests that amphiphile aggregates were sufficient by themselves to provide both compartmentalization and storage and transfer of compositional molecular information to subsequent generations during early abiogenesis. As postulated by Segré *et al.* (2000), this happens via compositional inheritance in multicomponent lipid vesicles to future generations brought about by growth-split cycles governed by mutual catalysis (Segré *et al.,* 2001b). The origin of these amphiphiles is also under debate, with hypotheses including both terrestrial and extraterrestrial sources (Deamer, 1985; Deamer and Pashley, 1989; Cronin, 1998; Hanczyc *et al.,* 2003; Bada, 2004; Thomas and Rana, 2007; Georgiou and Deamer, 2014; Mayer *et al.,* 2015). The question of how compositional inheritance in lipid assemblies transitions to polynucleotide-based inheritance is still wide open (Shenhav *et al.,* 2005). A third hypothesis suggests that compartmentalization emerged independently of information, and these two branches of evolution merged at some later date (Pulselli *et al.,* 2009; Regis, 2009). Such a development is less improbable than simultaneous emergence of both compartments and information-carrying polymers (Pulselli *et al.,* 2009). This view is supported by experimental findings that vesicles can grow and self-reproduce (Berclaz *et al.,* 2001). In a computational model, Fellermann and Solé (2007) argued that a dividing, cell-like structure can be composed of only a metabolism-container coupled system. This allows natural selection to be applied to an experimental system of self-reproducing vesicles (Božič and Svetina, 2004, 2007; Svetina, 2012) in terms of Oparin's principles, where simple self-replicating vesicles predate cells driven by informational molecules such as RNA, unknown at that time (Oparin, 1936). An alternative hypothesis suggests that a common chemistry generated precursors to all three components (compartmentalization, information, and metabolism) (Budin and Szostak, 2011; Blain and Szostak, 2014; Patel *et al.,* 2015).

The GARD model employed in our simulations is a formalism that follows the dynamic fate of amphiphile assemblies and enables asking specific questions about their capacities to portray certain life-like attributes. Early GARD simulations did not commit to a specific amphiphile chemistry (Segré *et al.,* 2001). In our previous study (Armstrong *et al.,* 2011), we invoked realistic amphiphilic molecules in our simulations, while considering which specific building blocks are best suited for simulations. Fatty acids are much simpler and more easily envisaged as prebiotic components (Segré *et al.,* 2001a). Whether phospholipids were present in the early prebiotic period is still a matter of debate. The possibility of prebiotically synthesized phospholipids was suggested by Hargreaves *et al.* (1977), but current consensus considers the early presence of phospholipids unlikely (Pohorille and Deamer, 2009). However, the importance of phospholipids in later evolution is indisputable, and there is much more thermodynamic and kinetic data available on phospholipids as compared with fatty acids, which is important for constructing a reasonable kinetic formalism from empirically derived parameters. Finally, when considering a role for GARD-invoked amphiphile structures as potential compartments, the fact that phospholipid vesicles are less leaky than those composed of fatty acids played a role in our decision.

In our previous publication (Armstrong *et al.,* 2011), we advanced a novel formalism based on a semi-empirical approach where data from published kinetic interactions of today's phospholipids with bilayer lipid vesicles of diverse compositions and properties were utilized to simulate the progression of such a vesicle system in the environment of infinite availability of lipid monomers. In contrast to most previous modeling and experimental studies that use amphiphile systems of limited number of components, we used a system that includes four classes of phospholipids: phosphatidylethanolamine (PE), phosphatidylserine (PS), phosphatidylcholine (PC), and sphingomyelin (SM), and cholesterol (CHOL), with a variety of acyl chain lengths and unsaturation levels comprising altogether 141 different lipid components. This component heterogeneity is much more realistic and can lead to different outcomes than simple systems with more limited numbers of component types (Szostak, 2011; Budin *et al.,* 2014). Nonlipid molecules present in the prebiotic milieu are likely to have influenced vesicle growth (see Adamala and Szostak, 2013), and future formalisms may include them.

In the present work, we extend our previous formalism to an environment of finite resources and examine the kinetics and properties of the resultant phospholipid vesicle system. These properties include high variability of vesicle composition and properties, critical importance of compositional heterogeneity, and vesicle-vesicle competition for diminishing resources. Additionally, these results inform efforts to construct *de novo* cells, as they predict the composition of cell membranes that will be stable given a particular environmental milieu.

## 2. Materials and Methods

### 2.1. Lipid vesicle simulations

Simulations of lipid vesicles were carried out by using a simulation environment that follows the kinetic parameters of our previous work (Armstrong *et al.,* 2011), as described in the Supplementary Material (available at http://online.liebertpub.com/doi/suppl/10.1089/ast.2016.1615), with two important modifications. First, the environment consisted of a limited number of lipid molecules (4.096 × 10^7^) for all 141 lipid types with a total environmental concentration of 1.41 × 10^−8^ *M* in a fixed volume. Lipids in the environment were depleted or added to as lipids entered or left vesicles during the simulation, but the total number of lipid molecules in the simulation remained constant. Second, all vesicles were tracked in the simulation, enabling vesicles to compete with each other for finite resources. Simulations ran for 300–1000 s and generally progressed to an average of 12 generations, with on the order of 2048 vesicles in each simulation at the end. The simulation environment noted the starting and ending vesicles for each division and stored the state of every vesicle every min(5 × 2^⌊*i*/50⌋^, 50) iterations, where *i* is the current iteration number. Vesicles divided once they reached 20,000 lipid molecules, and lipids in the parent vesicle were randomly assorted between each of the two progeny vesicles. Vesicle reproduction upon doubling was previously suggested as a result of mild shear forces acting on the growing vesicles (Zhu and Szostak, 2009; Budin and Szostak, 2011; Szostak, 2011). Such mild shear forces induce division without loss of the content and are common in natural environments (Zhu and Szostak, 2009).

It has been demonstrated, by using various experimental systems, that vesicles can grow and divide autonomously (Wick *et al.,* 1995; Hanczyc *et al.,* 2003; Takakura *et al.,* 2003, 2004; Thomas and Rana, 2007; Toyota *et al.,* 2008).

The rate of incorporation (or removal) of each lipid component into (or from) a vesicle is dependent upon the lipid's environmental concentration, base kinetic rate parameters, the average properties (curvature, charge, unsaturation, length, complex formation) of the vesicle into which the lipid is entering or leaving, the surface area of the vesicle, and the properties of that particular lipid molecule (length, type, unsaturation). For complete details of the kinetic formalism, see the Supplementary Material and Armstrong *et al.* (2011).

The simulations were carried out on a cluster in parallel using code written in Perl.

### 2.2. Plots and summary statistics

The states of the environment and vesicles were collected into an SQLite database, extracted using R scripts, and then plotted using ggplot2. Summary statistics were calculated by using linear least-squares models and *t* tests as appropriate.

## 3. Results

The simulations presented here are based on the R-GARD (Real Graded Autocatalysis Replication Domain) equation (Supplementary Material and Eq. 1), providing a quantitative description for the principle that kinetic parameters for lipid entry and exit depend on the current composition of vesicles they join. In this study, the simulations started with one seeded vesicle, and all progenies from each division were followed. The environment started with enough lipid material for 4096 lipid vesicles with average of 10,000 lipid molecules per vesicle (4.096 × 10^7^ lipid molecules overall).

**FIG. 1.**
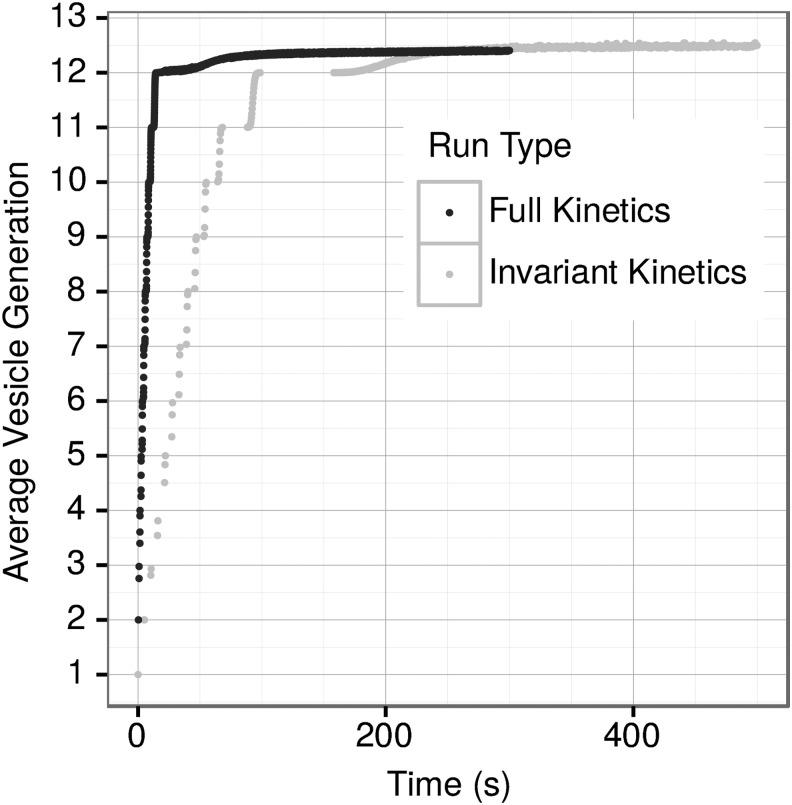
Average number of generations over time for full and invariant kinetics for multiple runs.

The average age in generations of all vesicles in the simulation when run with “full kinetics” (modification of lipid kinetics by the properties of the vesicles into which the lipids are entering or leaving) or with “invariant kinetics” (where the rates are not modified by the vesicle composition and properties) is shown in Fig. 1, which represents an average of multiple runs. Vesicles whose properties affect kinetic parameters grow and divide approximately an order of magnitude more rapidly than vesicles without modification due to vesicle properties. This is further illustrated in Table 1.

**Table 1.** Average Times for Vesicles to Reach an Average of 12 Generations in Simulations with Full Kinetics and Invariant Kinetics with All Lipids (All), and Missing a Single Lipid Type (*e.g.,* No CHOL)

*Formalism*	*Lipid types*	*Time (s)*	*SD*	*Runs*
Full kinetics	All	17.6	0.68	20
	No CHOL	34.6	9.52	6
	No PC	35.4	7.86	6
	No PE	22.5	1.76	6
	No PS	22.1	1.03	6
	No SM	13.7	0.21	6
Invariant kinetics	All	164.3	3.57	16
	No CHOL	267.5	2.69	6
	No PC	162.5	5.45	6
	No PE	138.0	2.26	6
	No PS	157.9	2.83	20
	No SM	155.6	4.17	6

Vesicles take 17.6 ± 0.7 s to reach an average generation of 12 in the full kinetics simulations, whereas vesicles take 164.3 ± 3.6 s in the invariant kinetics runs (Table 1). To explore unequal availability of different lipids, we ran the simulations without one lipid class (that is, without one PC, PE, PS, SM, or CHOL). Omitting CHOL decreases the time necessary to reach an average generation of 12 with full kinetics but increases the time by a factor of 2 with invariant kinetics (Table 1). Conversely, omitting PC increases the time to reach an average generation of 12 with full kinetics but has the opposite effect with invariant kinetics. Only minor effects on the overall time to reach an average generation of 12 were observed in cases of omitting PS, PE, or SM from the environment (Table 1).

**FIG. 2.**
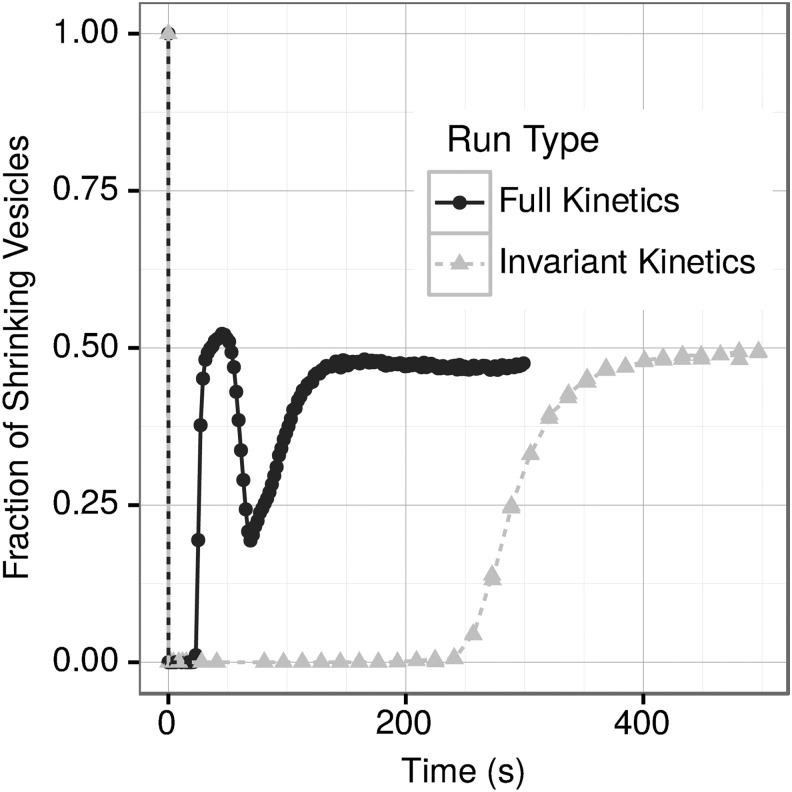
Average fraction of vesicles that are shrinking over time for the full and invariant kinetics simulations for multiple runs.

When our formalism was applied to a finite environment, we expected to find that vesicles with compositions that had less favorable kinetics would lose lipids to the environment. Those lipids lost to the environment would then be incorporated by vesicles with compositions that resulted in more favorable kinetics, providing evidence for competition among the vesicles. Figure 2 shows the fraction of vesicles that are shrinking over the total number of vesicles at a given time point in simulations with (“full kinetics”) and without (“invariant kinetics”)—an impact of vesicle properties upon kinetics. In both types of simulation, the initial vesicle shrinks slightly as a few lipids with high *k*_b_ are shed; then all vesicles in the simulation begin growing (0–15 s for full kinetics, 0–230 s for invariant kinetics, Fig. 2). After the initial shrinkage of the seed vesicle, the behavior of “full kinetics” is very different from that of the “invariant kinetics” simulations.

In simulations with full kinetics, a few distinct phases of the vesicle growth and division can be discerned (Fig. 2). After initial shrinkage, vesicles undergo an unrestricted growth phase until about 15 s. At this point, the depletion of free lipids from the environment results in an increase in the number of vesicles that are actually shrinking (15–47 s), until the number of shrinking vesicles is larger than the number of growing ones (Fig. 2, 47 s peak). However, at the same time, a significant proportion of the vesicles (approximately 45% at 47 s) are growing by incorporating the lipid molecules released by the shrinking vesicles, thus demonstrating competition among the vesicles for diminishing resources. The second growth phase (in this case due to the reduced fraction of the shrinking vesicles) occurs at 47–70 s. After 70 s, the system progresses toward the final steady state, reached at 170 s. The invariant kinetics formalism exhibits a simple kinetics behavior: unrestricted growth (0–230 s), transition to steady state (230–450 s), and steady state, reached at 450 s (Fig. 2).

**FIG. 3.**
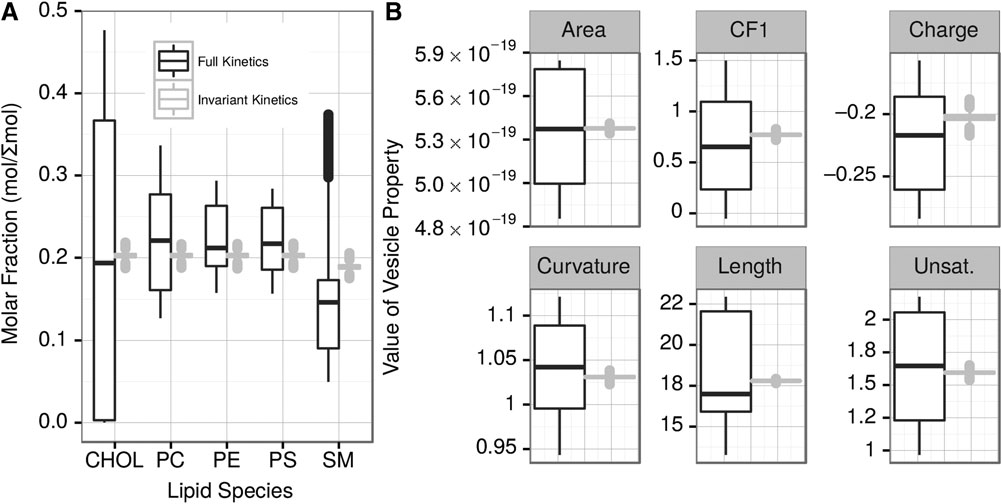
The molar fractions and variabilities of the vesicle lipid compositions (**A**) and values and variabilities of vesicle properties (**B**) with (full kinetics) and without (invariant kinetics) kinetic modifications at the end of the simulation for 20 full kinetics simulations and 16 invariant kinetics simulations. Area is shown in m^2^; the other properties are unitless.

Importantly, both vesicle composition and the resulting properties exhibit much higher variability in the full kinetics simulations (Fig. 3). Removing one lipid type from the environment in most cases did not result in significant changes; however, removing SM results in the second growth burst being much less pronounced (not shown).

**FIG. 4.**
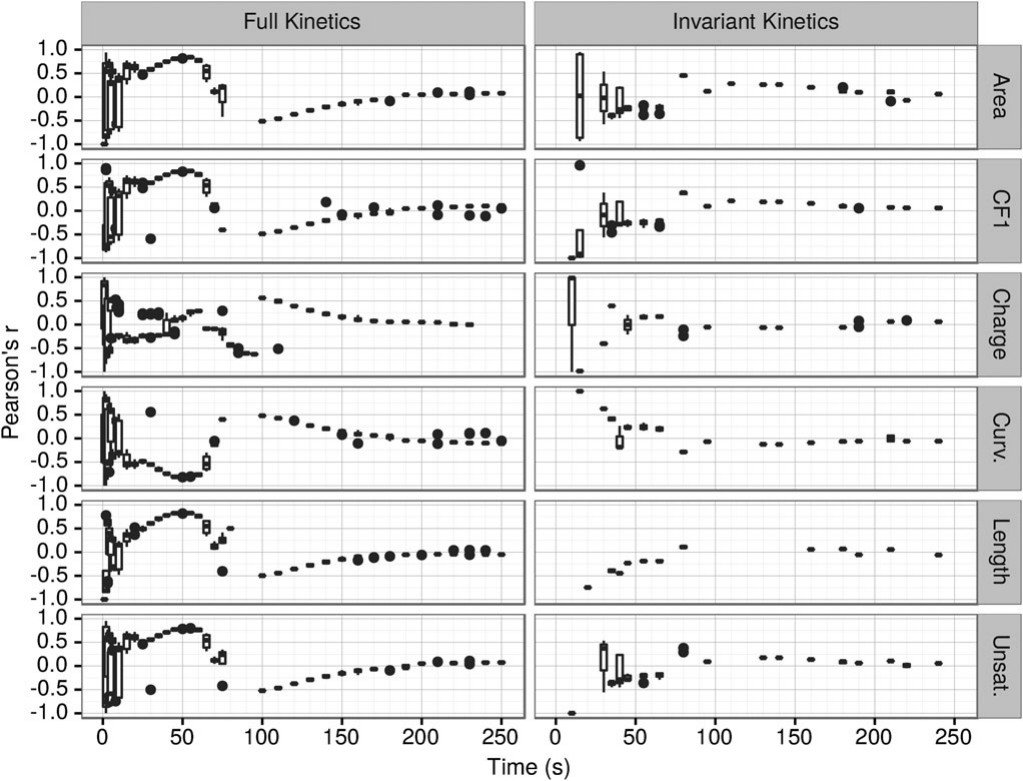
Pearson's *r* correlations between vesicle growth and vesicle properties.

**FIG. 5.**
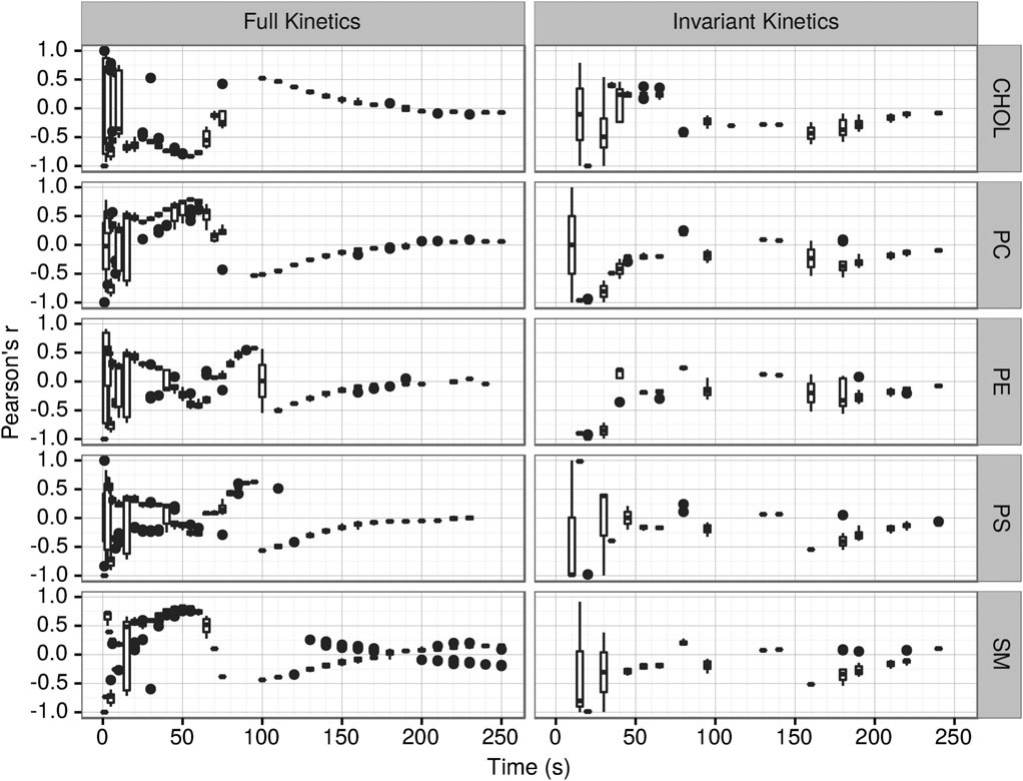
Pearson's *r* correlations between vesicle growth and lipid types.

The complexity of the kinetic rate modifying equations (Supplementary Material and Armstrong *et al.,* 2011) results in an accordingly complex correlation of vesicle growth with the vesicle composition and properties. Pearson correlation analysis of the time-dependent correlation of the vesicles' growth with their composition and properties (Figs. 4 and 5) mostly corresponds with growth and shrinkage phases identified on Fig. 2.

Distribution of the Pearson's product-moment correlation coefficient of significant (*p* ≤ 0.05) correlations between the rate of growth or shrinkage $\left( \frac { \Delta { \rm lipids } }  { \Delta { \rm s } } \right)$ and the properties of that vesicle over time for 10 full kinetics and 10 invariant kinetics simulations are shown on Fig. 4. In the full kinetics simulations, the correlations are relatively random for all vesicle properties (Fig. 4) and lipid classes (Fig. 5) in the first 20 s, with some simulations of the same variables showing positive correlation and others showing negative correlation as evidenced by the width of the distribution of values of PC at those times. In the time period between 35 and 70 s, more consistent correlations are seen throughout each simulation.

In the case of full kinetics, we observed in this time period (35–70 s) a strong positive correlation of the vesicle growth with the vesicle's length, unsaturation, area, and CF1 (Fig. 4, Table 2).

**Table 2.** Correlation of Vesicle Growth with Lipid Classes and Vesicle Properties at 51 s

	p	*Pearson's* r	*Adjusted* r^*2*^
Charge	1.86 × 10^−5^	0.18	0.03
Length	2.89 × 10^−52^	0.85	0.72
Unsaturation	6.23 × 10^−40^	0.82	0.67
Area	2.23 × 10^−60^	0.85	0.72
Curvature	2.45 × 10^−68^	−0.85	0.72
CF1	1.07 × 10^−67^	0.85	0.73
CHOL	1.60 × 10^−53^	−0.84	0.71
PE	9.61 × 10^−13^	−0.33	0.10
PS	1.86 × 10^−5^	−0.18	0.03
PC	1.63 × 10^−39^	0.77	0.60
SM	1.85 × 10^−64^	0.84	0.70

**FIG. 6.**
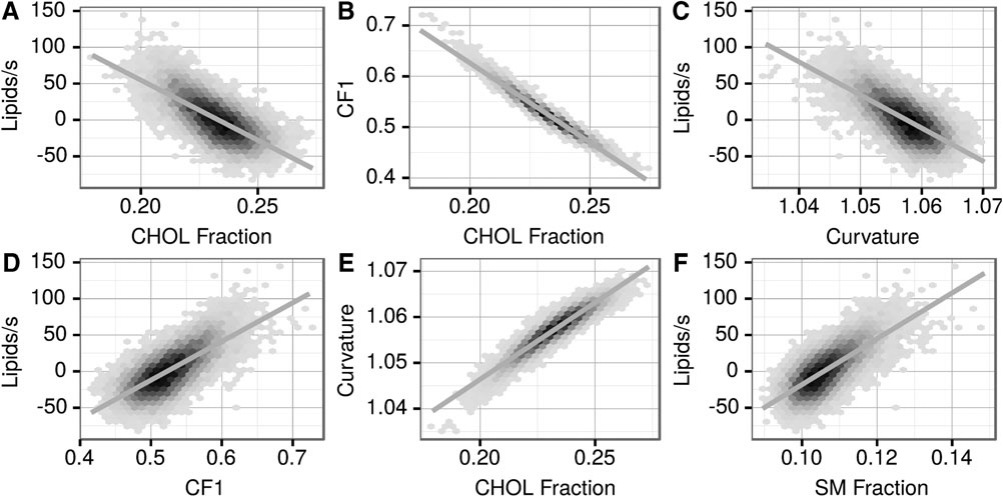
Correlation of the rate of growing or shrinkage of the vesicles at 37 s in lipids per second (competition/shrinking vesicles phase) with selected lipid classes and properties of the vesicles.

Conversely, curvature is negatively correlated with the vesicle growth (Figs. 4 and 6 and Table 2). Similarly, in most cases (PC, PS, SM), in the competition phase growth is positively correlated with the amount of lipid type in the vesicle (Fig. 5). However, this correlation is much less pronounced in the case of PE and is negative in the case of CHOL. In full kinetics after about 120–130 s, the correlations become too small (|*r*| < 0.2) to be meaningful.

Correlation of selected lipid types and properties with vesicle growth is shown on Fig. 6. Such correlations serve to understand the basic promoters and inhibitors of the vesicle growth.

Since CF1 is positively (Fig. 6D), and curvature negatively (Fig. 6C), correlated with growth, negative correlation with growth was expected and observed with the CHOL content (Fig. 6A). More typically and straightforwardly, the increased amount of a lipid correlated with growth (Fig. 6F).

**FIG. 7.**
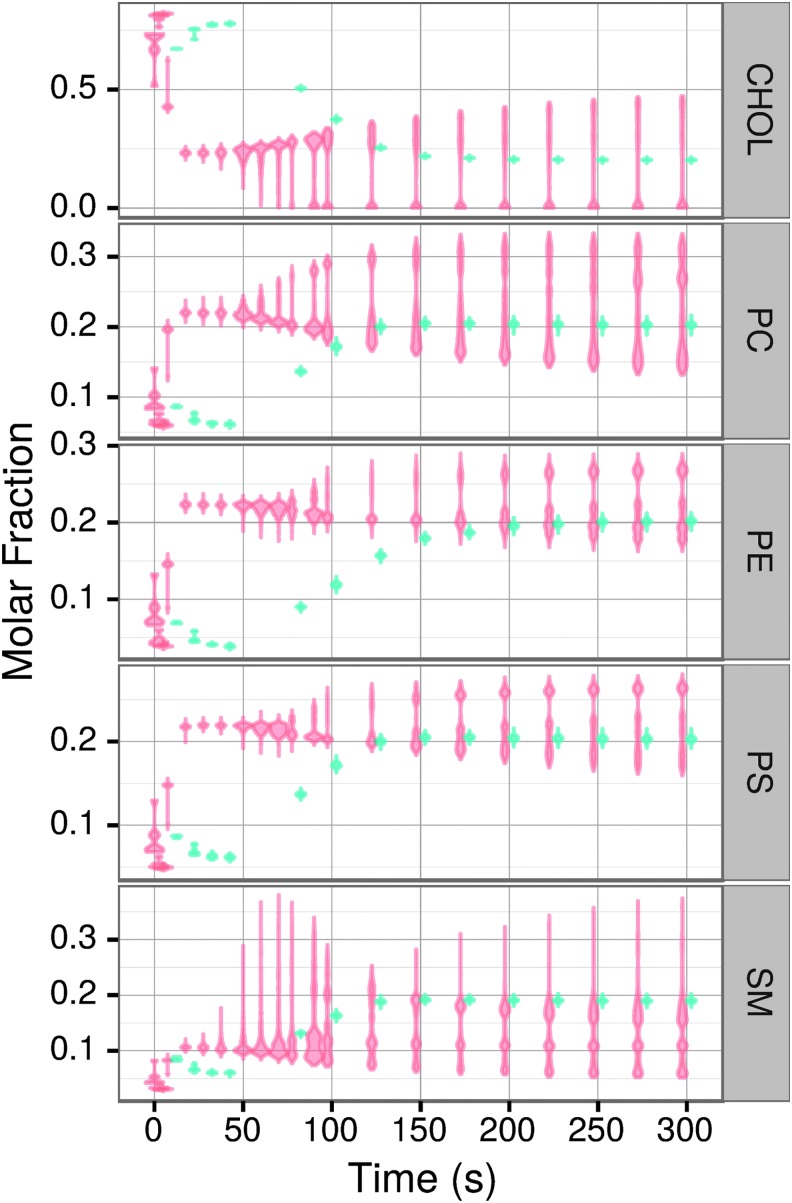
Violin plots of the time-dependent distribution of the fraction of lipid types contained within vesicles in full (orange) and invariant (blue) kinetics simulations.

**FIG. 8.**
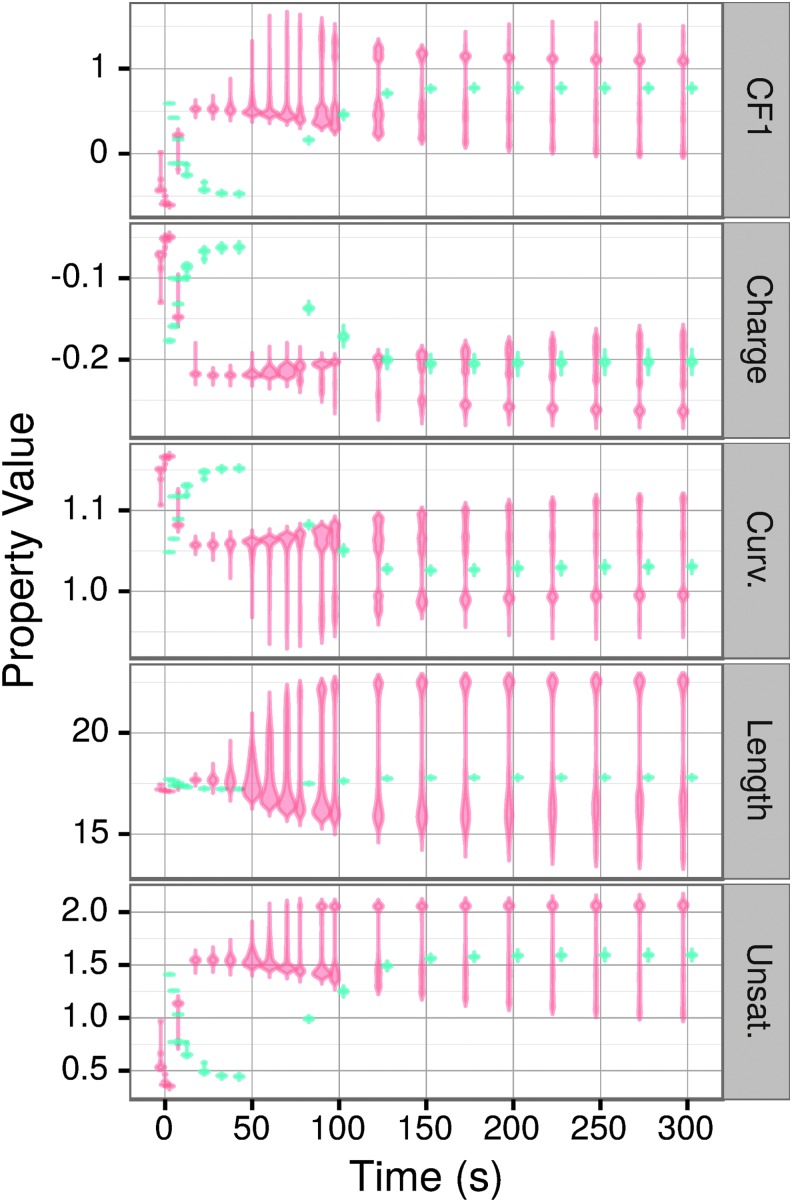
Violin plots of the time-dependent distribution of the properties of the vesicles in full (orange) and invariant (blue) kinetics simulations.

The changes in lipid composition and properties of the vesicles over time are shown in Figs. 7 and 8, respectively. A prominent feature for every lipid type is a much higher variability in vesicle composition at the final steady state in the full kinetics as compared to the invariant kinetics simulations. For example, CHOL molecular fractions range from nearly 0 in some vesicles to 0.5 in others in the full kinetics simulations, whereas the invariant kinetics simulations have all a CHOL fraction of 0.2 with only a small variation (Fig. 7). Other lipid types show more complicated distributions. Thus, in the full kinetics simulations, PC ranges from a molar ratio of approximately 0.13 to 0.35 in the steady state, but the molar ratios are not normally distributed about a mean: the allowed concentrations instead consist of three superimposed normal distributions at times greater than 130 s. As expected, given the changes in distribution of the molar ratios of lipid types over time, the distribution of the properties of vesicles shows similar complex patterns. Invariant kinetics simulations exhibit little variance in the distribution of vesicle composition (Fig. 7) or of vesicle properties (Fig. 8). Full kinetics simulations display a more complex behavior with the most prominent and important difference from invariant kinetics being an order of magnitude higher variability of the vesicles' composition and the resulting properties. In invariant kinetics simulations steady state is more slowly, consistent with the data shown in Fig. 1. Early in the simulation, vesicles are rich in CHOL because of its high *k*_f_. With time, all lipids in both the full and invariant kinetics converge to steady state values of about 0.2 mol/fraction, reflecting the corresponding ratios in the environment.

Accordingly, the properties' behavior corresponds to that of the lipid (or lipids) most prominently expressing a particular property. Charge is directly related to the concentration of PS, and its distribution mirrors that of PS; thus, at the start when the concentration of PS in the vesicles is relatively low (Fig. 7), the negative charge is low, too (Fig. 8). Curvature is primarily a function of the concentrations of CHOL, SM, and PS, and most closely follows the concentration of CHOL. This is also reflected in Fig. 8, where the curvature increases at the beginning as a consequence of the high CHOL fraction. In the case of CF1, we observed high initial CHOL concentration (CF1 = −1) and low initial SM concentration (CF1 = 3) (Fig. 7) that corresponded with initial low values of CF1 for vesicles (Fig. 8). The formalism preferentially selects for vesicles with similar-length lipids, and because CHOL only comes in one length, vesicles with higher CHOL content at early times preferentially contain lipids with lengths near 18 (Fig. 8). And finally, CHOL unsaturation of zero is reflected by the decrease of unsaturation at the beginning (Fig. 8).

**FIG. 9.**
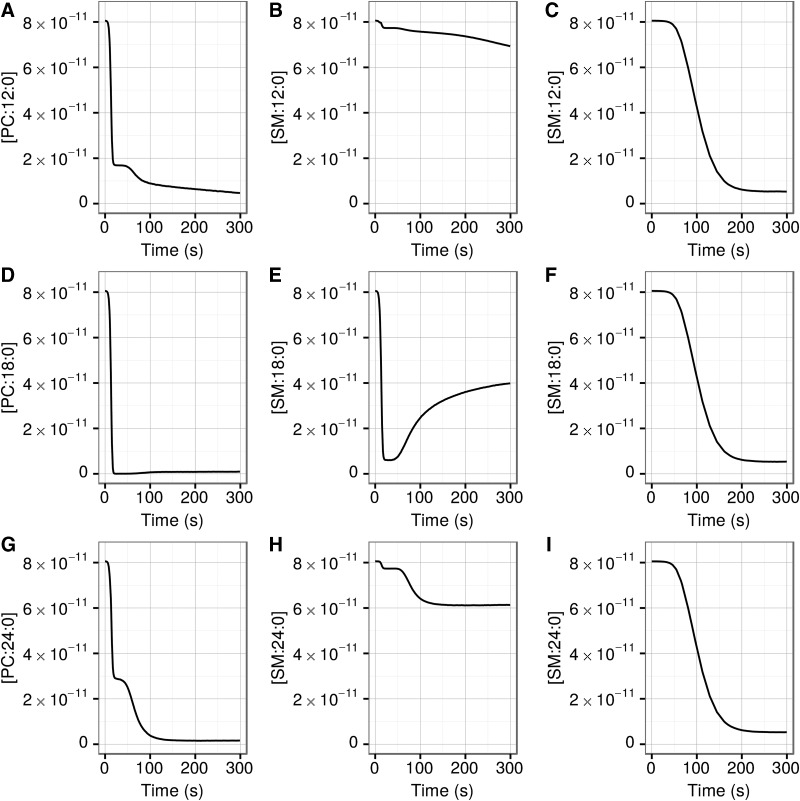
Time dependence of the environmental concentrations of selected lipids. *Y* axes are concentrations in *M.* Left and middle columns: full kinetics; right column: invariant kinetics simulations.

Time dependencies of the environment concentrations of full kinetics saturated PC (left) and SM (middle column) of the complete range of lengths, and of invariant kinetics SM (right), are shown in Fig. 9. All other lipids in the invariant kinetics simulations exhibited behavior similar to that of SM. Whereas SM in the invariant kinetics mode exhibits classic kinetics behavior, the behaviors of PC and, especially, SM in the full kinetics mode are considerably more complex. The first phase, unrestricted growth, is reflected by sharp decreases of all lipids in the environment. In most full kinetics cases, the next, competition/shrinking vesicle, phase (15–47 s) is reflected by a plateau of the environmental lipid concentrations, followed by further decrease corresponding to renewed lipid consumption by the vesicles. The plateau also corresponds to the increased fraction of the shrinking vesicles (Fig. 2). Some lipids (*e.g.,* PC 18:0, Fig. 9D) exhibit little or no plateau, because they were almost completely absorbed by the vesicles during the unrestricted growth phase due to their high *k*_f_.

Some SM components exhibit interesting behavior (see Fig. 9E) where they are released by vesicles into the environment after the first 50 s, corresponding to the competition/second growth burst. This is due to the large *k*_b_ of SM, which dominates the *k*_fadj_ for all SM that do not match the average length of the vesicle. All other lipids exhibit behavior similar to PC. The final consequence of high *k*_b_ is that all other non-SM lipids are virtually completely consumed by the vesicles (see Fig. 9A, 9D, 9G for PC), but much of SM remains in the environment (Fig. 9B, 9E, 9H).

**FIG. 10.**
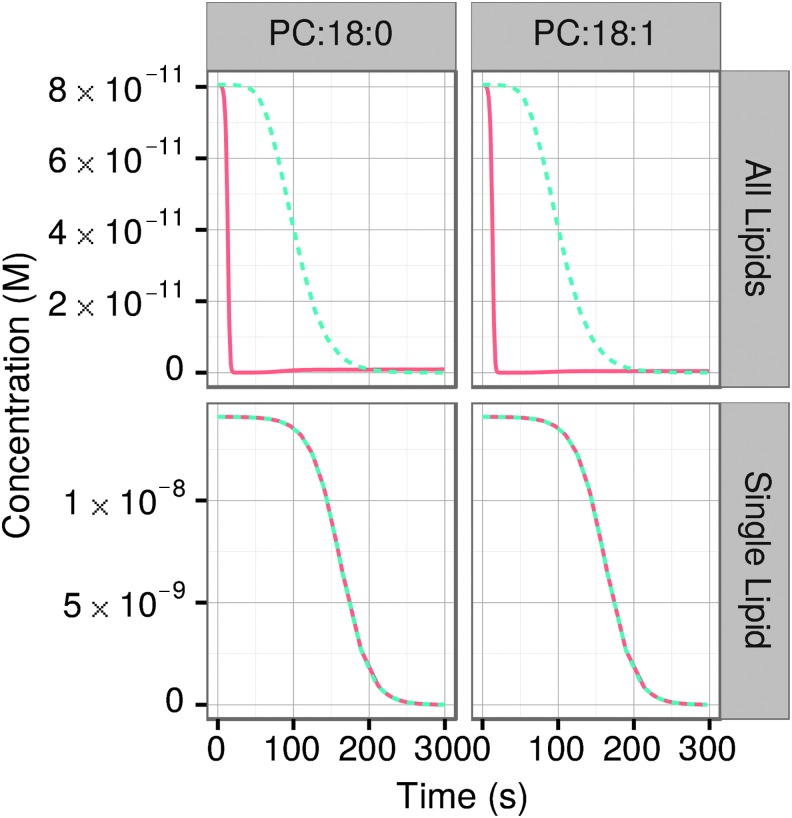
The role of lipid heterogeneity. Comparison of runs with all lipids or a single lipid (*i.e.,* DOPC or DPPC) and full (orange solid line) or invariant (blue dotted line) kinetics.

We have further investigated the role of lipid component heterogeneity in the complex behavior of the lipid system at full kinetics. When only one component is present, the resulting kinetics are essentially identical for full and invariant kinetics at all times, in contrast with the multicomponent runs (Fig. 10). Schrum *et al.* (2010) pointed out that prebiotic vesicles were likely composed of complex mixtures of amphiphiles and noted that amphiphilic molecules found in meteorites (Deamer, 1985; Deamer and Pashley, 1989) and those synthesized under simulated prebiotic conditions (McCollom *et al.,* 1999; Dworkin *et al.,* 2001; Rushdi and Simoneit, 2001) are highly heterogeneous.

**FIG. 11.**
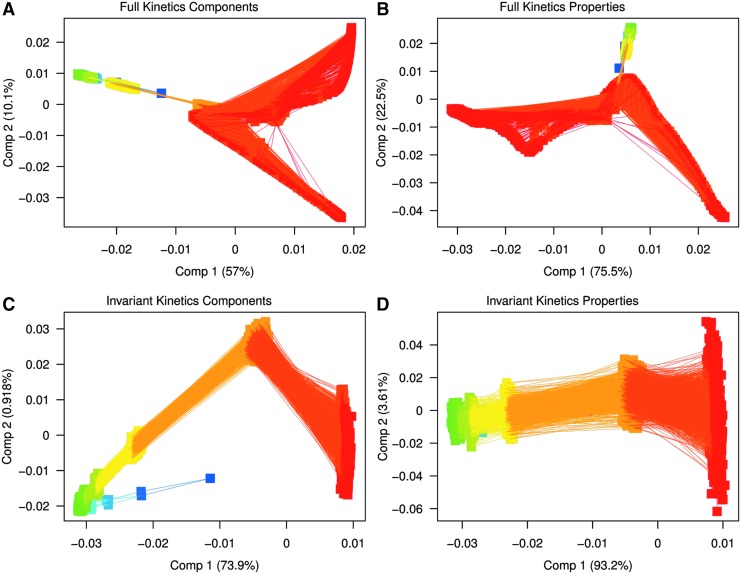
PCA plots of lipid components (**A**, **C**) and properties (**B**, **D**) for full (A, B) and invariant (C, D) kinetics. The color scheme progresses from the first generations (blue to green) to the last (red).

The principal components analysis (PCA) plots (Fig. 11) also demonstrate higher variability of both vesicle properties and compositions in the case of full kinetics. This is the best seen in the variation along the first two components occupied by the final generation (in red) of full kinetics for the lipid components (range of 0.03 in component 1 and 0.06 in component 2 for full kinetics [Fig. 11A] vs. essentially constant in component 1 and 0.03 in component 2 for invariant kinetics [Fig. 11C]). Similarly, in the case of vesicle properties, the variation for component 1 for full kinetics is 0.055 (Fig. 11B), as compared to the essentially constant at 0.01 for the invariant kinetics (Fig. 11D). Component 2 in the full kinetics properties varies by 0.05 (Fig. 11B), and in the invariant kinetics by 0.1 (Fig. 11D), agreeing with Figs. 7 and 8 that demonstrate high variability of the composition and properties of the vesicles in the full kinetics mode.

## 4. Discussion

In this study, we have extended our formalism, which includes modification of kinetic parameters of lipid monomer–vesicle interaction by the composition and the resulting properties of the vesicles (Armstrong *et al.,* 2011) to an environment with finite resources. Competition for finite resources among vesicles introduces selective pressure, which, in combination with the kinetic formalism, is entirely responsible for the behavior of vesicles in this system.

Extensive comparisons of the full kinetics and invariant kinetics formalisms demonstrate crucial differences, essential for considering this relatively simple lipid vesicle system as a model of the prebiotic evolution. Specifically, we found that in the full kinetics system vesicles replicate almost an order of magnitude faster than in the invariant kinetics system. Furthermore, whereas the invariant kinetics system exhibits simple classical behavior of initial growth, followed by decay to final steady state as the resources diminish, the behavior of the full kinetics system is much more complex and exhibits the following distinct phases.

(1) *Unrestricted growth phase* until about 15 s. This phase is similar to the general behavior of the system with unlimited resources described in our previous publication (Armstrong *et al.,* 2011). (2) *Inter-vesicle competition phases*. (2.1) *Shrinking vesicles*. The depletion of free lipids in the environment results in an increase in the fraction of vesicles that are actually shrinking at 15–47 s, until the number of shrinking vesicles is larger than the number of growing ones. Since most of the resources at this point are consumed, the vesicles that are growing are obtaining material from other, shrinking vesicles. (2.2) *Second growth burst* (in this case due to the reduced fraction of shrinking vesicles) occurs at 47–70 s. (3) *Decay to steady state,* 70–170 s, and (4) *Final steady state,* reached at ∼170 s. In contrast, the invariant kinetics formalism results in a simple “classical” kinetics behavior: unrestricted growth (0–230 s), decay to steady state (230–450 s), and finally steady state (reached at 450 s).

Our formalism, which is based on the modification of the kinetic rates by the compositions and thus the properties of the vesicles, differs in essential aspects from previously published studies. A computational system to study the structural properties and dynamic behavior of lipid vesicle populations was developed by Mavelli and Ruiz-Mirazo (2010) and tested experimentally. There are some substantial differences with our formalism. We seed the simulations with one vesicle, as compared to 50 vesicles as described by Mavelli and Ruiz-Mirazo (2010). This allowed us to simulate the entire course of a vesicle population from a single vesicle to the maximal number of 4096 vesicles. In the process, we demonstrated that appreciable vesicle compositional variability is spontaneously generated even when initiating the simulation with only one composition, stemming from stochastic factors in the vesicle division as a “natural” built-in variability generator. Using terminology of Damer and Deamer (2015), in our case “Each vesicle represents a protocell, an ‘experiment’ in a natural version of combinatorial chemistry.” Damer and Deamer (2015) also considered cycles of hydration and dehydration, whereas in our system a constant excess of water is present. Finally, in our formalism, kinetics strongly depends on the vesicle structure, whereas the only bilayer structure parameter taken into account by Mavelli and Ruiz-Mirazo (2010) was elastic/bending state of the membrane as a result of osmotic stress.

One of the most distinguishing processes we identified that is relevant for evolution is intervesicle competition. Vesicle competition was also demonstrated experimentally by Shirt-Ediss *et al.* (2014) where vesicles were “stealing” lipids from each other as a function of their lipid composition and membrane asymmetry. Shirt-Ediss *et al.* (2014) did account for lipid composition by altering the kinetic rates of lipid monomers that enter and leave vesicles, although in a way different from ours. The “direct effect” described by Shirt-Ediss *et al.* (2014) makes a general assumption that the acyl tails of phospholipids have a high affinity for packing closer to each other and increasing bilayer order, thus making the exit of the simple lipids more difficult, which is also explicitly included in our formalism. In addition to this, we also take into account a number of other effects, including charge, shape, complex formation, and the degree of unsaturation of the phospholipids (Armstrong *et al.,* 2011, and Supplementary Material).

Competition between vesicles was also previously experimentally observed among fatty acid vesicles prepared under different physical conditions, for example, swollen or isotonic (Chen *et al.,* 2004), again different from our system where the simulations start with one vesicle and competition is observed among its progenies. Chen *et al.* (2004) showed that vesicles with high internal osmotic pressure (caused in that particular case by incorporated RNA or sucrose) can acquire lipids from isotonic vesicles. However, their system included at most two membrane lipid components. In geological settings, a phase of crowded protocells enables competition and selection similar to the behavior of the lipid vesicle system in our simulations (Damer, 2016).

We have concentrated on resource availability in this study because it is one of the main factors that determines the population dynamics (Pekkonen *et al.,* 2013). Our results emphasize the critical importance of heterogeneity in lipid component types. In our previous study, we noted the importance of such heterogeneity for high variability of the vesicles under the conditions of unlimited resources (Armstrong *et al.,* 2011). In the current study, we demonstrate that systems composed of only one lipid type replicate much slower and do not exhibit the complex behavior that is observed with multicomponent lipid mixtures, including an absence of different phases, such as vesicle shrinking/lipid redistribution, second growth burst phase, and vesicle competition among progenies. Indeed our simulations with only one lipid component present gave results identical for both full and invariant kinetics. An approach that allows a certain degree of heterogeneity—“controlled complexity”—is more realistic and potentially more useful for the prebiotic studies than oversimplified homogeneous systems (Szostak, 2011).

Based on the observation that the inclusion of phospholipids into fatty acid vesicles results in the combined vesicles acquiring material faster and losing it slower, Budin and Szostak (2011) suggested that the “evolution of phospholipid membranes could thus have been a deterministic outcome of intrinsic physical processes and a key driving force for early cellular evolution.” However, because the properties of a vesicle are necessarily determined by its composition, which can change rapidly, vesicles transmit their compositional information content to subsequent generations with much more stochasticity than template-directed processes, such as RNA replication. Furthermore, the full kinetics system grew an order of magnitude faster than the invariant kinetics system. Finally, multicomponent vesicles grow almost an order of magnitude faster than simple vesicles with only one component, thus providing a selective advantage (under competition) for including multiple complementary components.

Our analyses explore two antipodal cases: in one, the rates of lipid joining and leaving do depend on vesicle composition; in another, they do not. We show that only in the first case a complex chain of events is observed along the time axis (complex kinetics), including intervesicle competition for diminishing resources and a growth burst driven by better-adapted vesicles, a behavior that is similar to evolution in later forms of early life. In contrast, replication is much slower and vesicle variability is smaller in simulations where vesicle composition does not alter kinetic parameters. Both replication speed and variability between vesicles are determinants of evolutionary behavior. All of these findings become clearer in the present paper, which explores for the first time the effects on GARD evolutionary dynamics in an environment with a finite supply of lipids.

Some of the main conclusions of our *in silico* results are testable experimentally. Among them, changes in the environmental concentrations of lipids (Fig. 9) where there are distinct qualitative and quantitative differences between the full and invariant kinetics formalisms, including in the most interesting manifestation: the consumption followed by a partial release back to the environment of some lipid constituents, the most pronounced with SM. Furthermore, the importance of a large number of component types for replication speed can be tested by comparing behaviors of a simple one-component system with a multicomponent one to see which system incorporates lipids more rapidly. We predict that a single-component system will behave similarly to the invariant kinetics one (*cf.* Fig. 10). Perhaps the most interesting phenomenon predicted here is a large proportion of shrinking vesicles at specific stages of the process. The growth or shrinkage of vesicles can be directly observed, for example, by using a membrane-staining fluorescent dye and time-lapse confocal microscopy, as described by Hardy *et al.* (2015). The conditions used can be adjusted to correspond to the specific questions asked, for example to the physical environment of conditions on the prebiotic Earth.

In summary, utilizing this formalism where the kinetics constants are affected by the vesicle composition and properties, we have demonstrated *in silico* that a lipid vesicle system of a high number of component types, composed of extant phospholipids and cholesterol, exhibits prebiotic properties such as replication, competition, and high variability of vesicle composition and properties, which are critical for evolution. These *in silico* predictions are amenable to testing by physical experiment. In addition to physical experiments, future developments of this model include the addition of biopolymers that can interact with the simulated membranes and external selective pressures, such as thermal stress, hydration cycles, and ion availability.

## Supplementary Material

Supplemental data

## Abbreviations Used

CHOL: cholesterol
PC: phosphatidylcholine
PCA: principal components analysis
PE: phosphatidylethanolamine
PS: phosphatidylserine
R-GARD: Real Graded Autocatalysis Replication Domain
SM: sphingomyelin
