# Supplementary material for: Replication of Simulated Prebiotic Amphiphilic Vesicles in a Finite Environment Exhibits Complex Behavior That Includes High Progeny Variability and Competition
Source: Astrobiology. 2018 Apr 1;18(4):419–30. doi: 10.1089/ast.2016.1615 (PMC5910049; doi:10.1089/ast.2016.1615)
Supplement: Supplemental data [file Supp_Data.pdf]

Appendix 1: Kinetic formalism for R-GARD Simulations

1 State Equation

For a system with monomers ($_{\text{monomer}}$) and a vesicle ($_{\text{vesicle}}$), the change in concentration of the ith component of a lipid vesicle per change in time ($d\left[C_{i_{\text{vesicle}}}\right]/dt$) can be described by a modification of the basic mass action law:

$$\frac{d\left[C_{i_{\text{vesicle}}}\right]}{dt} = k_{\mathrm{f}i}k_{\mathrm{f}i\mathrm{adj}}\left[C_{i_{\text{monomer}}}\right]\left[S_{\text{vesicle}}\right] - k_{\mathrm{b}i}k_{\mathrm{b}i\mathrm{adj}}\left[C_{i_{\text{vesicle}}}\right] \tag{1}$$

The base forward kinetic parameter for the ith component is $k_{\mathrm{f}i}$ and is dependent on the particular lipid type (phosphatidylcholine (PC), phosphatidylserine (PS), sphingomyelin (SM), etc.). The forward adjustment parameter, $k_{\mathrm{f}i\mathrm{adj}}$, is based on the properties of the vesicle and the specific component (type, length, unsaturation, etc.) (see Eq. (2) and Section 2). $\left[C_{i_{\text{monomer}}}\right]$ is the molar concentration of monomer of the ith component. $\left[S_{\text{vesicle}}\right]$ is the surface area of the vesicle per volume. The base backwards kinetic parameter for the ith component is $k_{\mathrm{b}i}$ and its adjustment parameter $k_{\mathrm{b}i\mathrm{adj}}$ (see Eq. (8) and Section 2). $\left[C_{i_{\text{vesicle}}}\right]$ is the molar concentration of the ith component in the vesicle.

1.1 Per-Lipid Kinetic Parameters

Each of the 5 lipid types has different kinetic parameters; where available, these were taken from literature (Table S1).

Type	k_f $\left(\frac{\mathrm{m}}{\mathrm{s}}\right)$	k'_f $\left(\frac{1}{\mathrm{Ms}}\right)$	k_b (s^{-1})	Area $\left(\mathrm{\AA}^2\right)$	Charge	CF1	Curvature
PC	9.75×10^{-3}	3.7×10^{6}	2×10^{-5}	63	0	2	0.8
PS	9.75×10^{-3}	3.7×10^{6}	1.25×10^{-5}	54	-1	0	1
CHOL	0.13	5.1×10^{7}	2.8×10^{-4}	38	0	-1	1.21
SM	9.75×10^{-3}	3.7×10^{6}	3.1×10^{-3}	61	0	3	0.8
PE	6.06×10^{-3}	2.3×10^{6}	1×10^{-5}	55	0	0	1.33

Table S1: Kinetic parameters and molecular properties of lipid types

1.1.1 k_f for lipid types

$k_{\mathrm{f}_\mathrm{PC}}$ was measured by Nichols [1] and was found to be $3.7 \times 10^6\ \frac{1}{\mathrm{Ms}}$ by the partitioning of 1-palmitoyl-2-6-[(7-nitro-2-1,3-benzoxadiazol-4-yl)amino]hexanoyl-*sn*-glycero-3-phosphocholine (P-C_6-NBD) between 1,2-dioleoyl-*sn*-glycero-3-phosphocholine (DOPC) vesicles and water. As similar references are not available for SM or PS, we assume that they have the same k_f. For cholesterol (CHOL), no direct measurement of k_f is available, however, Estronca *et al.* [2] measured the transfer of dehydroergosterol (DHE) from bovine serum albumin (BSA) to lipid unilamellar vesicles (LUVs), and found a k_f of $5.1 \times 10^7\ \frac{1}{\mathrm{Ms}}$. We assume that this value is close to that of CHOL, and use it for $k_{\mathrm{f}_\mathrm{CHOL}}$. In the case of phosphatidylethanolamine (PE), Abreu *et al.* [3] measured the association of n-(7-nitro-2-1,3-benzoxadiazol-4-yl)amino-1,2-dimyristoylphosphatidylethanolamine (NBD-DMPE) with 1-palmitoyl-2-oleoyl-*sn*-glycero-3-phosphocholine (POPC) LUVs and found a value for k_f of $2.3 \times 10^6\ \frac{1}{\mathrm{Ms}}$. These three authors used a slightly different kinetic formalism ($\frac{d[A_v]}{dt} = k'_\mathrm{f}[A_m][L_v] - k_\mathrm{b}[A_v]$), so we correct their values of k_f by multiplying by the surface area of a mole of lipids.

1.1.2 k_b for lipid types

Wimley & Thompson [4] measured the half time for the exchange of [^{3}H]1,2-dimyristoylphosphatidylcholine (DMPC) between LUVs at 30°C, and found it to be 9.6 hr. As this is a first order reaction, and the primary limiting step in exchange is lipid desorption, k_b for DMPC is $k_{\mathrm{b}_\mathrm{PC}} = \frac{\log 2}{9.6 \times 60 \times 60} \approx 2.01 \times 10^{-5}\mathrm{s}^{-1}$. We assume that k_b for SM is the same as for PC. To estimate the k_b of PE and PS, we used the data from Nichols & Pagano [5] who measured the rate of exchange of the fluorescent label

C_6-NBD attached to different lipid species. Although the values of k_b are different for the labeled and unlabeled lipids, we assume that the ratios of the kinetics constants for the lipid types are the same. Furthermore we assume that phosphatidylglycerol (PG) behaves similarly to PS. Thus, we can determine the k_b of PE and PS from the already known k_b of PC. For C_6-NBD labeled PC, Nichols & Pagano [5] obtained a k_b of 0.89 min^{-1}, PE of 0.45 min^{-1} and PG of 0.55 min^{-1}. Assuming the ratios of the rate of exchange are the same for unlabeled lipids and labeled lipids, we can determine the k_b of PE and PS from the already known k_b of PC [4]. Calculating the ratio, this leads us to $k_{b_{PE}} = \frac{k_{b_{PC}} \times \mathrm{PE}}{\mathrm{PC}} \approx \frac{2\times10^{-5}\,\mathrm{s}^{-1}\times 0.45\,\mathrm{min}^{-1}}{0.89\,\mathrm{min}^{-1}} \approx 1.01 \times 10^{-5}$ s^{-1} and likewise, $k_{b_{PS}} \approx 1.24 \times 10^{-5}$ s^{-1}. The k_b of SM was determined using the work of Bai & Pagano [6], who measured spontaneous transfer of C_5-DMB-SM and C_5-DMB-PC from donor and acceptor vesicles, finding 3.4×10^{-2} s^{-1} and 2.2×10^{-3} s^{-1} respectively; using the ratio of k_b of C_5-DMB-SM to the k_b of C_5-DMB-PC times the k_b of PC ($\frac{3.4\times10^{-2}\mathrm{s}^{-1}}{2.2\times10^{-3}\mathrm{s}^{-1}} \approx 2.01 \times 10^{-5}\mathrm{s}^{-1}$), we obtain $k_{b_{SM}} \approx 3.1 \times 10^{-4}$. In the case of CHOL, Jones & Thompson [7] measured the $t_{1/2}$ of [^{3}H] transfer from POPC vesicles and found it to be 41 minutes, leading to a $k_{b_{CHOL}} = \frac{\log 2}{41\times60} \approx 2.82 \times 10^{-4}$ s^{-1}.

1.1.3 Headgroup Surface Area for lipid types

Different lipids have different headgroup surface areas, which contributes to $[S_{\mathrm{vesicle}}]$. Smaby *et al.* [8] measured the surface area of POPC with a Langmuir film balance, and found it to be 63 Å^2 at 30 $\frac{\mathrm{mN}}{\mathrm{m}}$. Molecular dynamic simulations found an area of 54 Å^2 for 1,2-dipalmitoyl-*sn*-glycero-3-[Phospho-L-Serine] (DPPS)[9, 10], which is in agreement with the experimental value of 56 Å^2 found using a Langmuir balance by Demel *et al.* [11]. Shaikh *et al.* [12] measured the area of SM using a Langmuir film balance, and found it to be 61 Å^2. Using ^{2}H NMR, Thurmond *et al.* [13] found the area of 1,2-dipalmitoyl-*sn*-glycero-3-phosphoethanolamine (DPPE)-d_{62} to be 55.4 Å^2. Robinson *et al.* [14] found an area for CHOL of 38 Å^2 using molecular dynamic simulations.

1.1.4 Complex Formation 1

Thomas & Poznansky [15] found that SM significantly decreases the rate of cholesterol transfer from PC, while PE and PS have no effect at physiologically significant levels. We attribute this to the formation of complexes between SM and CHOL, which retards the rate of efflux of CHOL from the membrane. Similar complexes exist between PC and CHOL, where it was shown that two

CHOL molecules complex with one PC [16–19]. It has also been shown that SM binds more CHOL molecules than PC [20]. We assume that three CHOL complex with one SM, leading to CF1 values of 3, 2, and -1 for SM, PC, and CHOL, respectively.

1.1.5 Curvature

We used the formulation for curvature of Israelachvili *et al.* [21], or $S = \frac{v}{l_c a}$, where S is the curvature, which ranges from 0 to ∞, l_c is the critical length of the acyl chain, v is the volume of the lipid, and a is the area of the head group. Kumar [22] found a value for S of 1.33 for PE, 1.21 for CHOL, and 0.8 for diC_{16} PC. We assume that PS has neutral curvature (value of 1), and that SM has the same curvature as PC (0.8). It should also be noted that in reality the curvature parameter changes with length, but at longer chain lengths, is effectively constant; in the current model, curvature is held constant for each species.

2 Kinetic adjustments

In the mass action equation used in the formalism, both the forward and backwards kinetic constants (k_{f} and k_{b}) are adjusted (by $k_{\mathrm{f}i\mathrm{adj}}$ and $k_{\mathrm{b}i\mathrm{adj}}$) to reflect the properties of the vesicle and the properties of the monomer entering or exiting the vesicle.

2.1 Forward Kinetic Adjustments

The forward rate constant adjustment, $k_{\mathrm{f}i\mathrm{adj}}$ takes into account unsaturation (un_{f}), charge (ch_{f}), curvature (cu_{f}), length (l_{f}), and complex formation ($CF1_{\mathrm{f}}$), each of which is modified depending on the specific component and the vesicle into which the component is entering; the final $k_{\mathrm{f}i\mathrm{adj}}$ is the product of the adjustments made separately for each property.

$$k_{\mathrm{f}i\mathrm{adj}} = un_{\mathrm{f}} \cdot ch_{\mathrm{f}} \cdot cu_{\mathrm{f}} \cdot l_{\mathrm{f}} \cdot CF1_{\mathrm{f}} \tag{2}$$

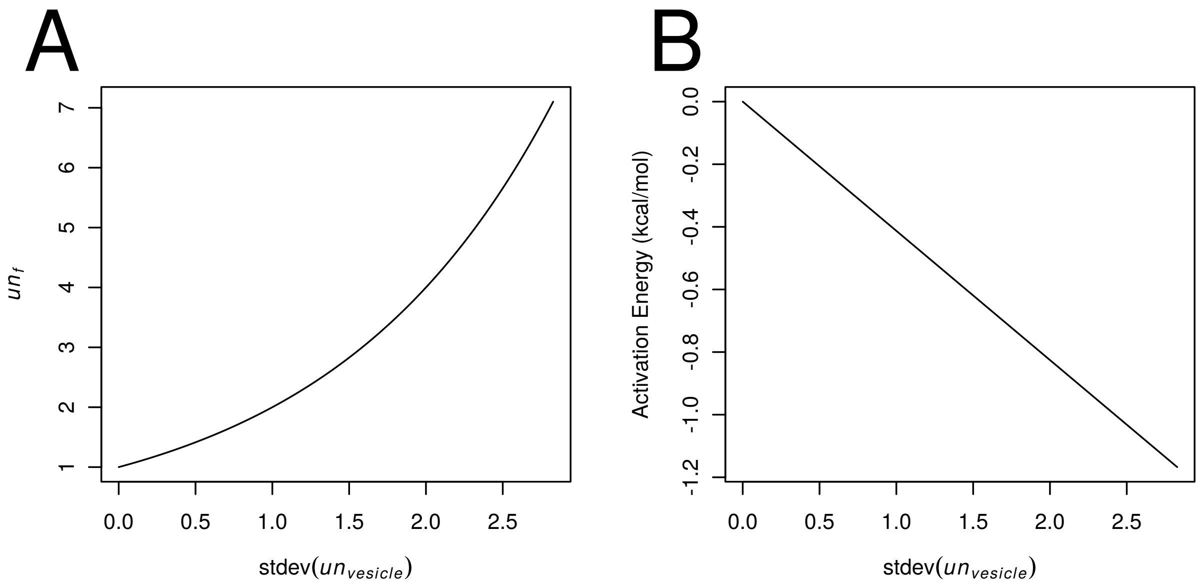

Figure S1: Change in unsaturation forward (un_f) (A) or activation energy in $\frac{\mathrm{kcal}}{\mathrm{mol}}$ (B) versus the standard deviation of the unsaturation of the vesicle (stdev (un_vesicle)).

2.1.1 Unsaturation Forward

In order for a lipid to be inserted into a membrane, a void has to be formed for it to fill. Voids can be generated by the combination of unsaturated and saturated lipids forming heterogeneous domains. Void formation is increased when the unsaturation of lipids in the vesicle is widely distributed; in other words, the insertion of lipids into the membrane is greater when the standard deviation of the unsaturation is larger. Assuming that an increase in width of the distribution linearly decreases the free energy of activation, the un_f parameter must follow $a^{\mathrm{stdev}(un_\mathrm{vesicle})}$ where $a > 1$, so a convenient starting base for a which leads to a reasonable change in Eyring activation energy is 2:

$$un_\mathrm{f} = 2^{\mathrm{stdev}(un_\mathrm{vesicle})} \tag{3}$$

The mean stdev (un_vesicle) in our simulations is around 1.5, which leads to a $\Delta\Delta G^\ddagger$ of $-0.619\frac{\mathrm{kcal}}{\mathrm{mol}}$, and a total range of possible values depicted in Fig. S1.

2.1.2 Charge Forward

A charged lipid such as PS approaching a vesicle with an average charge of the same sign will experience repulsion, whereas those with different signs will experience attraction, the degree of which is dependent upon the charge of the monomer and the average charge of the vesicle. If either the vesicle or the monomer has no charge, there should be no effect of charge upon the rate. This leads us to the following equation, $a^{-\langle ch_v \rangle ch_m}$ (which is similar to the numerator of Coulomb's law), where $\langle ch_v \rangle$ is the average charge of the vesicle, and ch_m is the charge of the monomer. If either $\langle ch_v \rangle$ or ch_m is 0, the adjustment parameter is 1 (no change), whereas it decreases if both are positive or negative, as the product of two real numbers with the same sign is always positive. The base for a (60) was chosen ad hoc to correspond to a maximum of about $0.5\frac{\text{kcal}}{\text{mol}}$ change in activation energy for the common case, resulting in the following equation:

$$ch_\text{f} = 60^{-\langle ch_v \rangle ch_m} \tag{4}$$

The most common $\langle ch_v \rangle$ is around -0.165, which leads to a range of $\Delta\Delta G^\ddagger$ from $-0.402\frac{\text{kcal}}{\text{mol}}$ to $0\frac{\text{kcal}}{\text{mol}}$, and the total range of possible values seen in Fig. S2.

2.1.3 Curvature Forward

Curvature is a measure of the intrinsic propensity of specific lipids to form micelles (positive curvature), inverted micelles (negative curvature), or planar sheets (neutral curvature) [21]. In this formalism, curvature is measured as the ratio of the volume of the lipid to the area of the head times the length of the lipid ($S = \frac{v}{l_c a}$), so negative curvature is bounded by $(0, 1)$, neutral curvature is 1, and positive curvature is bounded by $(1, \infty)$. The curvature can be transformed using log, which has the property of making the range of positive and negative curvature equal, and distributed about 0.

As in the case of unsaturation, void formation is increased by the presence of lipids with mismatched curvature. Thus, a larger distribution of curvature in the vesicle increases the rate of lipid insertion into the vesicle. However, a component with curvature cu^{-1} will cancel out a component with curvature cu, so we have to log transform (turning these into $-\log cu$ and $\log cu$), then take the absolute value ($\log cu$ and $\log cu$), and finally measure the width of the distribution, which in the case of exactly mismatched curvatures is 0. Thus, by using the log transform to make the range of

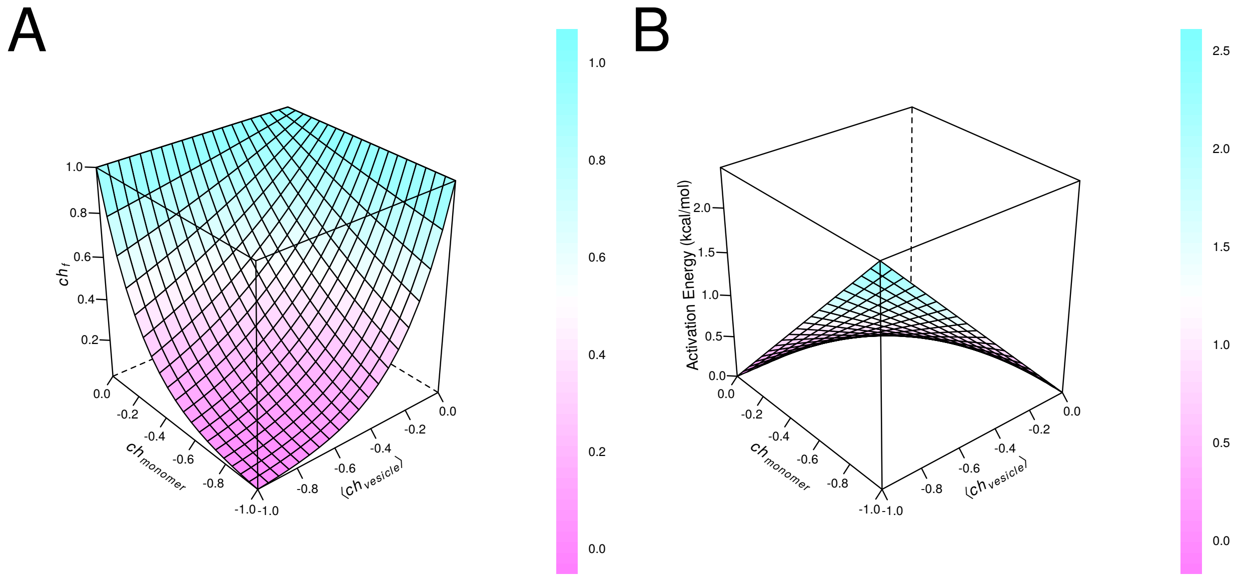

Figure S2: Change in charge forward (ch_f) (A) or activation energy in $\frac{\mathrm{kcal}}{\mathrm{mol}}$ (B) versus the charge of the monomer entering (ch_monomer) and the average charge of the vesicle ($\langle ch_\mathrm{vesicle} \rangle$).

the lipid curvature equal between positive and negative, and taking the average to cancel out exactly mismatched curvatures, we come to an equation with the shape $a^{\langle \log cu_\mathrm{vesicle} \rangle}$. An ad hoc base for a which for the common case is 10, yielding:

$$cu_\mathrm{f} = 10^{|\langle \log cu_\mathrm{monomer} \rangle| \mathrm{stdev} |\log cu_\mathrm{vesicle}|} \tag{5}$$

The most common $|\langle \log cu_v \rangle|$ is around 0.013, which with the most common stdev $\log cu_\mathrm{vesicle}$ of 0.213 leads to a $\Delta\Delta G^\ddagger$ of $-0.038 \frac{\mathrm{kcal}}{\mathrm{mol}}$. This is a consequence of the relatively matched curvatures in our environment. The full range of cu_f values possible are shown in Fig. S3.

2.1.4 Length Forward

As in the case of unsaturation, void formation is easier when vesicles are made up of components of mismatched lengths. Thus, when the width of the distribution of lengths is larger, the forward rate should be greater as well, leading us to an equation of the form $x^{\mathrm{stdev} l_\mathrm{vesicle}}$, where $\mathrm{stdev} l_\mathrm{vesicle}$ is the

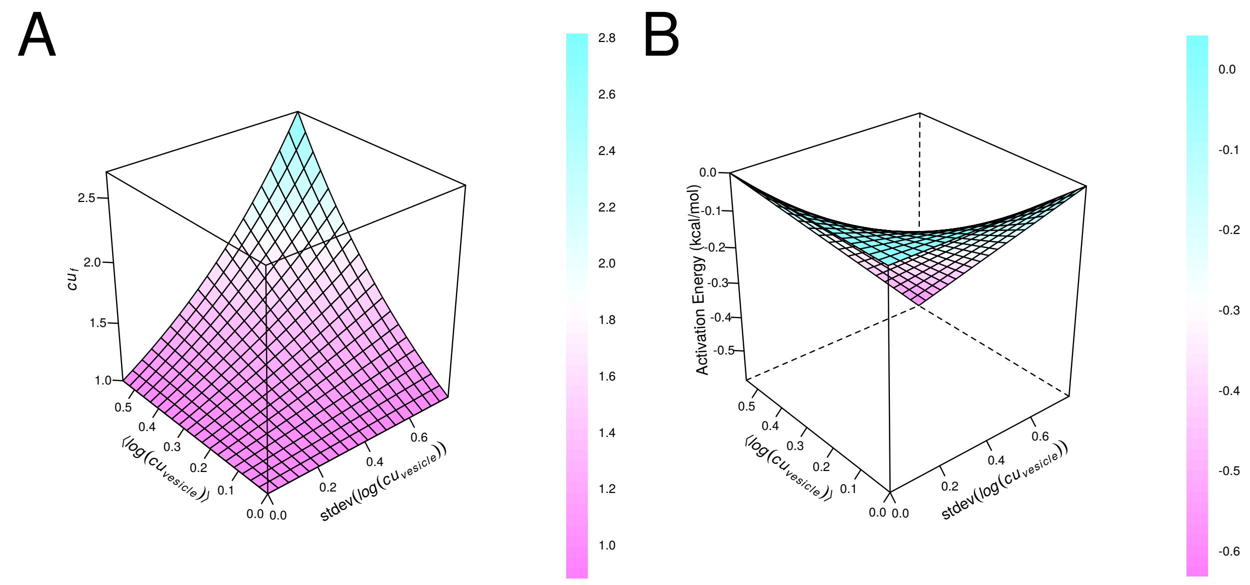

Figure S3: Change in curvature forward (cu_f) (A) or activation energy in $\frac{\mathrm{kcal}}{\mathrm{mol}}$ (B) versus the standard deviation of the log of the curvature of the vesicle (stdev $(\log cu_\mathrm{vesicle})$) and the mean of the log of the curvature of the vesicle ($\langle \log cu_\mathrm{vesicle} \rangle$).

standard deviation of the length of the components of the vesicle, which has a maximum possible value of 6.03 and a minimum of 0 in this set of simulations. Based on activation energy, a reasonable base for x is 2, leading to:

$$l_\mathrm{f} = 2^{\mathrm{stdev} l_\mathrm{vesicle}} \tag{6}$$

The most common stdevl_vesicle is around 3.4, which leads to a $\Delta\Delta G^\ddagger$ of $-1.4\frac{\mathrm{kcal}}{\mathrm{mol}}$.

2.1.5 Complex Formation

There is no contribution of complex formation to the forward reaction rate in the current formalism.

$$CF1_\mathrm{f} = 1 \tag{7}$$

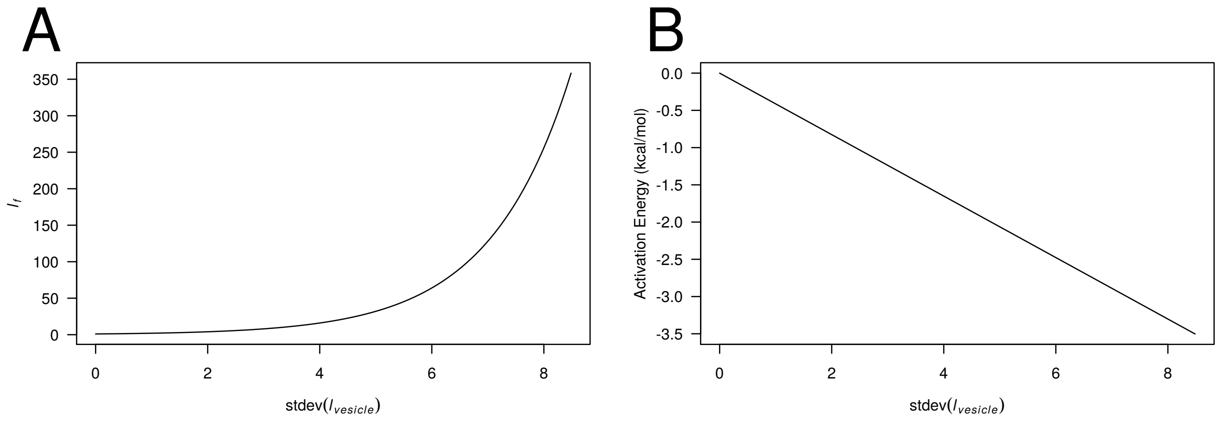

Figure S4: Change in length forward (l_{f}) (A) or activation energy in $\frac{\mathrm{kcal}}{\mathrm{mol}}$ (B) versus the standard deviation of the length of the vesicle (stdev (l_{vesicle})).

2.2 Backward adjustments ($k_{\mathrm{b}i\mathrm{adj}}$)

Just as the forward rate constant adjustment $k_{\mathrm{f}i\mathrm{adj}}$ does, the backwards rate constant adjustment $k_{\mathrm{b}i\mathrm{adj}}$ takes into account unsaturation (un_{b}), charge (ch_{b}), curvature (cu_{b}), length (l_{b}), and complex formation ($CF1_{\mathrm{b}}$), each of which is modified depending on the specific component and the vesicle from which the component is exiting:

$$k_{\mathrm{b}i\mathrm{adj}} = un_{\mathrm{b}} \cdot ch_{\mathrm{b}} \cdot cu_{\mathrm{b}} \cdot l_{\mathrm{b}} \cdot CF1_{\mathrm{b}} \tag{8}$$

2.2.1 Unsaturation Backward

Unsaturation also influences the ability of a lipid molecule to leave a membrane. If a molecule has an unsaturation level which is different from the surrounding membrane, it will be more likely to leave the membrane. The more different the unsaturation level is, the greater the propensity for the lipid molecule to leave. However, a vesicle with some unsaturation (eg. average unsaturation of 2) is more favorable for lipids with more unsaturation (eg. 3) than lipids with equivalently less unsaturation (eg. 1), so the difference in energy between unsaturation is not linear. Therefore, an equation with the shape $x^{\left|y^{-\langle un_{\mathrm{vesicle}}\rangle} - y^{-un_{\mathrm{monomer}}}\right|}$, where $\langle un_{\mathrm{vesicle}}\rangle$ is the average unsaturation of the vesicle and un_{monomer} is the unsaturation of the monomer, allows for increasing the efflux of molecules from membranes

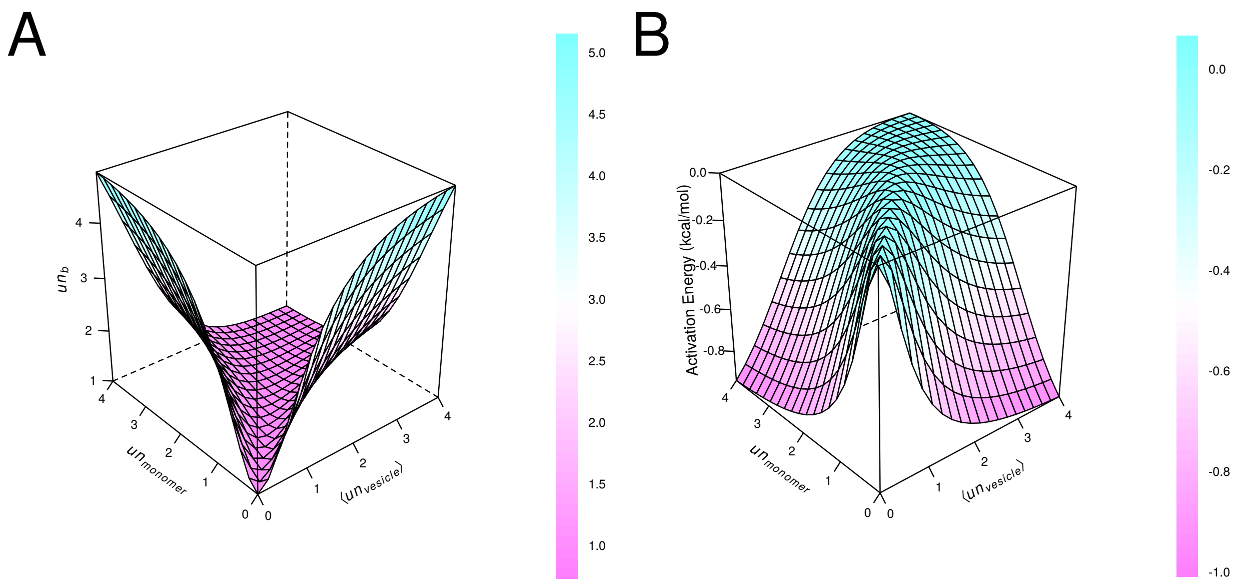

Figure S5: Change in unsaturation backward (un_b) (A) or activation energy in $\frac{\text{kcal}}{\text{mol}}$ (B) versus the unsaturation of the monomer leaving (un_monomer) and the average unsaturation of the vesicle ($\langle un_\text{vesicle}\rangle$).

where they strongly mismatch, while allowing vesicles with greater unsaturation to tolerate greater unsaturation mismatch between monomers and the vesicle. The bases x and y were chosen ad hoc to produce reasonable Eyring energies of activation over the range of unsaturations expected, leading to:

$$un_\text{b} = 7^{1-\left(20\left(2^{-\langle un_\text{vesicle}\rangle}-2^{-un_\text{monomer}}\right)^2+1\right)^{-1}} \tag{9}$$

The most common $\langle un_\text{vesicle}\rangle$ is around 1.7, which leads to a range of $\Delta\Delta G^\ddagger$ from $-0.818\frac{\text{kcal}}{\text{mol}}$ for monomers with 0 unsaturation to $-0.268\frac{\text{kcal}}{\text{mol}}$ for monomers with 4 unsaturations. See Fig. S5 for the full range of possible values.

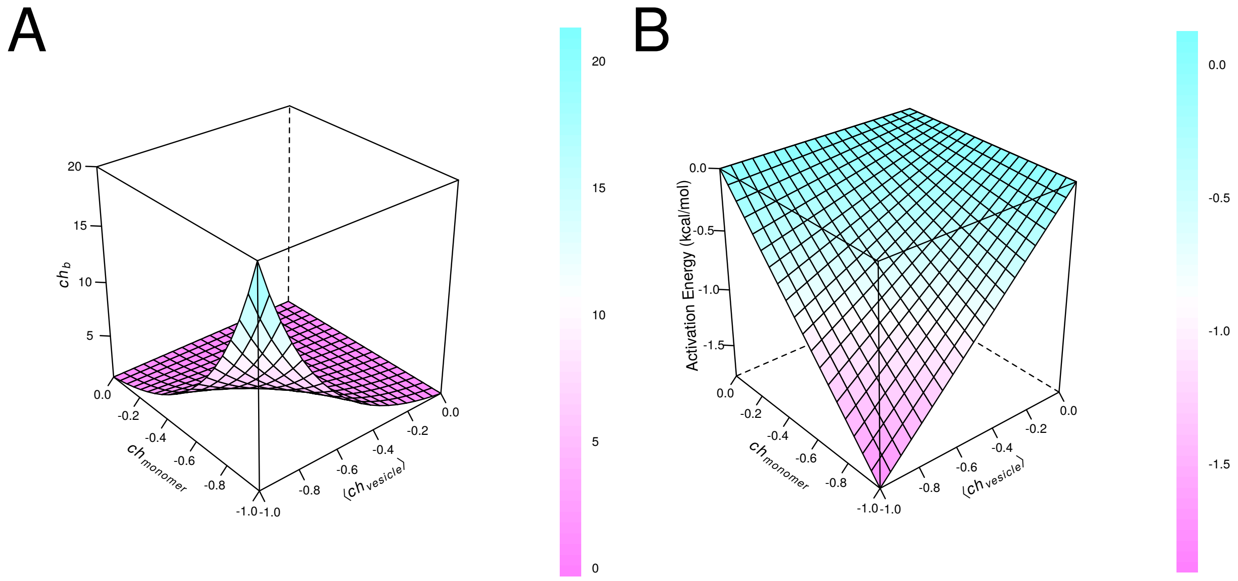

Figure S6: Change in charge backward (ch_b) (A) or activation energy in $\frac{\mathrm{kcal}}{\mathrm{mol}}$ (B) versus the charge of the monomer leaving (ch_monomer) and the average charge of the vesicle ($\langle ch_\mathrm{vesicle} \rangle$).

2.2.2 Charge Backwards

As in the case of monomers entering a vesicle, opposites attract. Monomers leaving a vesicle leave faster if their charge has the same sign as the average charge vesicle. An equation of the form $ch_\mathrm{b} = a^{\langle ch_v \rangle ch_m}$ is then appropriate, and using a base of $a = 20$ yields:

$$ch_\mathrm{b} = 20^{\langle ch_v \rangle ch_m} \tag{10}$$

The most common $\langle ch_v \rangle$ is around -0.164, which leads to a range of $\Delta\Delta G^\ddagger$ from $-0.293 \frac{\mathrm{kcal}}{\mathrm{mol}}$ for monomers with charge -1 to $0 \frac{\mathrm{kcal}}{\mathrm{mol}}$ for monomers with charge 0. See Fig. S6 for the full range of possible values of ch_b.

2.2.3 Curvature Backwards

The less a monomer's intrinsic curvature matches the average curvature of the vesicle in which it is in, the greater its rate of efflux. If the curvatures match exactly, cu_f needs to be one. To map negative

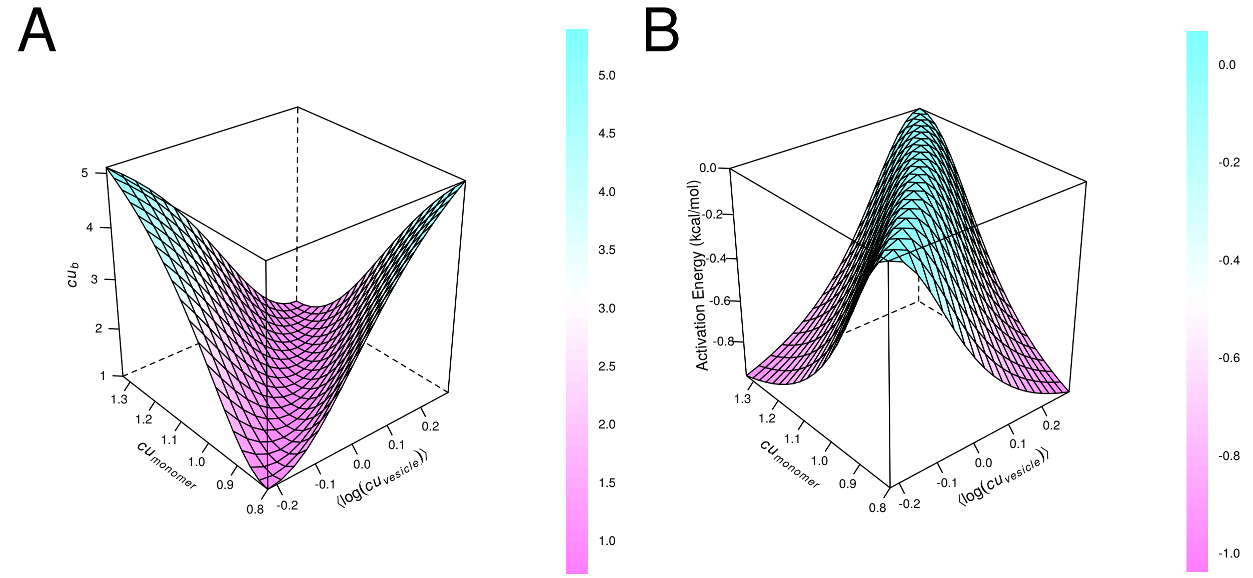

Figure S7: Change in curvature backward (cu_{b}) (A) or activation energy in $\frac{\mathrm{kcal}}{\mathrm{mol}}$ (B) versus the curvature of the monomer leaving (ch_{monomer}) and the average of the log of the curvature of the vesicle ($\langle \log cu_{\mathrm{vesicle}} \rangle$).

and positive curvature to the same range, we also need take the logarithm. Positive ($cu > 1$) and negative ($0 < cu < 1$) curvature lipids cancel each other out, resulting in an average curvature of 1; the average of the log also has this property (average curvature of 0). Increasing mismatches in curvature increase the rate of efflux, but asymptotically. An equation which satisfies these criteria has the form $cu_{\mathrm{f}} = a^{1-\left(b(\langle \log cu_{\mathrm{vesicle}} \rangle - \log cu_{\mathrm{monomer}})^2+1\right)^{-1}}$. An alternative form would use the absolute value of the difference, however, this yields a cusp and sharp increase about the curvature equilibrium. We have chosen bases of $a = 7$ and $b = 20$ ad hoc, based on activation energy considerations.

$$cu_{\mathrm{b}} = 7^{1-\left(20(\langle \log cu_{\mathrm{vesicle}} \rangle - \log cu_{\mathrm{monomer}})^2+1\right)^{-1}} \tag{11}$$

The most common $\langle \log cu_{\mathrm{vesicle}} \rangle$ is around -0.013, which leads to a range of $\Delta\Delta G^{\ddagger}$ from $-0.543\frac{\mathrm{kcal}}{\mathrm{mol}}$ for monomers with curvature 0.8 to to $0\frac{\mathrm{kcal}}{\mathrm{mol}}$ for monomers with curvature near 1 $-0.698\frac{\mathrm{kcal}}{\mathrm{mol}}$ for monomers with curvature 1.3. The full range of values possible for cu_{b} are shown in Fig. S7.

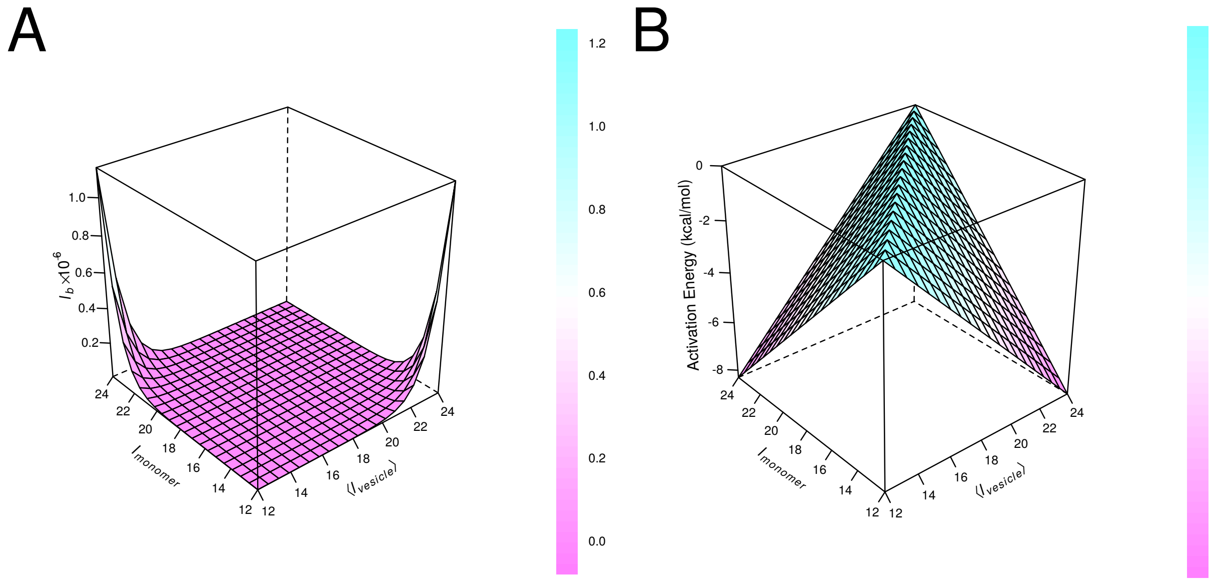

Figure S8: Change in length backwards ($l_\mathrm{b} \times 10^{-6}$) (A) or activation energy in $\frac{\mathrm{kcal}}{\mathrm{mol}}$ (B) versus the length of the monomer leaving (l_{monomer}) and the average length of lipids in the vesicle ($\langle l_{\mathrm{vesicle}} \rangle$).

2.2.4 Length Backwards

In a model membrane, the dissociation constant increases by a factor of approximately 3.2 per carbon decrease in acyl chain length [1]. Unfortunately, the experimental data known to us only measures the effect of chain lengths less than or equal to the bulk lipid, not the effect of lengths exceeding it, and is only known for one bulk lipid component (DOPC). We assume then, that the increase is in relationship to the average vesicle, and that lipids with larger acyl chain length will also show an increase in their dissociation constant.

$$l_\mathrm{b} = 3.2^{|\langle l_{\mathrm{vesicle}} \rangle - l_{\mathrm{monomer}}|} \tag{12}$$

The most common $\langle l_{\mathrm{vesicle}} \rangle$ is around 17.75, which leads to a range of $\Delta\Delta G^{\ddagger}$ from $-3.98\frac{\mathrm{kcal}}{\mathrm{mol}}$ for monomers with length 12 to $0\frac{\mathrm{kcal}}{\mathrm{mol}}$ for monomers with length near 18 to $-4.33\frac{\mathrm{kcal}}{\mathrm{mol}}$ for monomers with length 24. The full range of possible values of l_b are shown in Fig. S8.

2.2.5 Complex Formation Backward

Complex formation ($CF1$) describes the interaction between CHOL and PC or SM, where PC or SM protects the hydroxyl group of CHOL from interactions with water [16–19]. PC ($CF1 = 2$) can interact with two CHOL, and SM ($CF1 = 3$) with three CHOL ($CF1 = -1$). If the average of $CF1$ is positive (excess of SM and PC with regards to complex formation), components with negative $CF1$ (CHOL) will be retained. If average $CF1$ is negative, components with positive $CF1$ are retained. An equation which has this property is $CF1_{\text{b}} = a^{\langle CF1_{\text{vesicle}}\rangle CF1_{\text{monomer}} - |\langle CF1_{\text{vesicle}}\rangle CF1_{\text{monomer}}|}$, where difference of the exponent is zero if the average $CF1$ and the $CF1$ of the component have the same sign, or double the product if the signs are different. Based on activation energy considerations, we took an ad hoc base for a as 1.5.

$$CF1_{\text{b}} = 1.5^{\langle CF1_{\text{vesicle}}\rangle CF1_{\text{monomer}} - |\langle CF1_{\text{vesicle}}\rangle CF1_{\text{monomer}}|} \tag{13}$$

The most common $\langle CF1_{\text{vesicle}}\rangle$ is around 0.925, which leads to a range of $\Delta\Delta G^{\ddagger}$ from $0.447\frac{\text{kcal}}{\text{mol}}$ for monomers with complex formation -1 to $0\frac{\text{kcal}}{\text{mol}}$ for monomers with complex formation 2 to $0\frac{\text{kcal}}{\text{mol}}$ for monomers with complex formation 0. The full range of possible values for $CF1_{\text{b}}$ are depicted in Fig. S9.

3 Simulation Methodology

3.1 Overall Architecture

The simulations are currently run using a program written in perl with various modules to handle the subsidiary parts. It produces output for each generation, including the step immediately preceding and immediately following a vesicle split (and optionally, each step) that is written to a state file which contains the state of the vesicle, environment, kinetic parameters, program invocation options, time, and various other parameters necessary to recreate the state vector at a given time. This output file is then read by a separate program in perl to produce different output which is generated out-of-band; output which includes graphs and statistical analysis is performed using R [23] (and various grid graphics modules) called from the perl program.

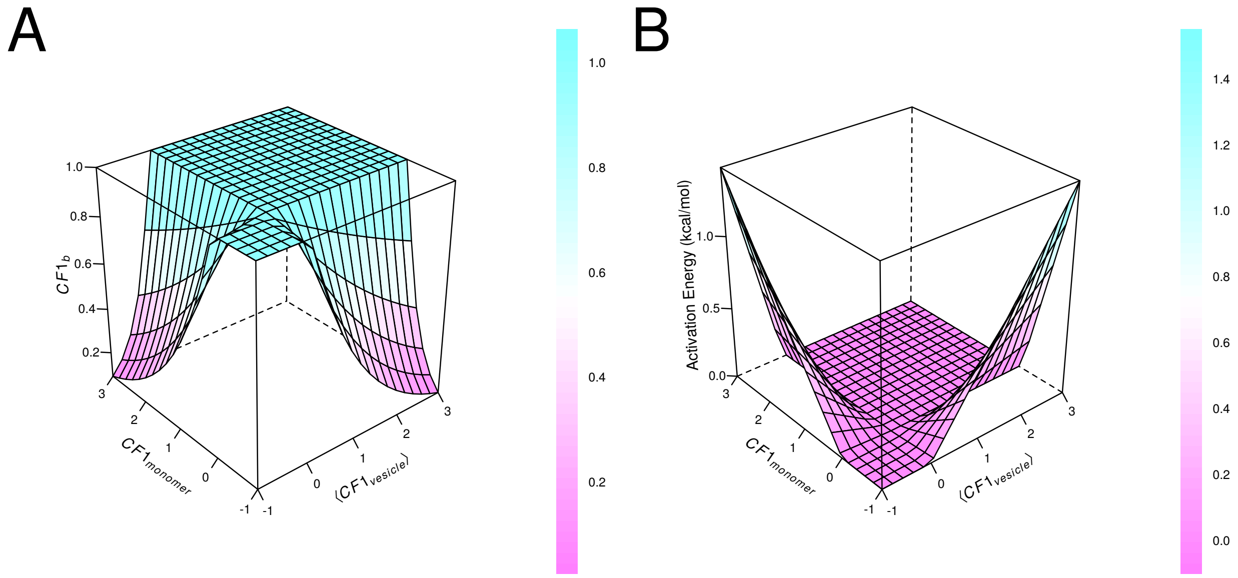

Figure S9: Change in complex formation 1 backwards ($CF1_{\mathrm{b}}$) (A) or activation energy in $\frac{\mathrm{kcal}}{\mathrm{mol}}$ (B) versus the complex formation of the monomer leaving ($CF1_{\mathrm{monomer}}$) and the average complex formation of the vesicle ($\langle CF1_{\mathrm{vesicle}} \rangle$).

The separation of simulation and output generation allows refining output, and simulations performed on different versions of the underlying code to be compared using the same analysis methods and code. It also allows later simulations to be restarted from a specific generation, utilizing the same environment.

Random variables of different distributions are calculated using Math::Random (0.71), which is seeded for each run using entropy from the linux kernel's urandom device.

Code is available upon request.

3.2 Environment Creation

3.2.1 Components

The environment contains concentrations of components. In the current set of simulations, there are 141 different possible components, consisting of PC, PE, PS, SM, and CHOL, with all lipids except

for CHOL having 5 possible unsaturations ranging from 0 to 4, and 7 lengths from $12, 14, ..., 22$ $(7 \cdot 5 \cdot 4 + 1 = 141)$. In cases where the environment has less than the maximum number of components, the components are selected in order without replacement from a randomly shuffled deck of components (with the exception of CHOL) represented once until the desired number of unique components is obtained. CHOL is overrepresented 35 times to be at the level of other lipid types, ensuring that the probability of CHOL being absent in the environment is the same as the probability of any one of the other lipid types (PS, PE, etc.) being absent. This reduces the number of simulations with a small number of components which are devoid of CHOL.

3.2.2 Concentrations

Once the components of the environment have been selected, their concentrations are determined. In experiments where the environmental concentration is the same across all lipid components, the concentration is set to 10^{-10} M. In other cases, the environmental concentration is set to a random number from a gamma distribution with shape parameter 2 and an average of 10^{-10} M. The base concentration (10^{-10} M) can also be altered at the initialization of the experiment to specific values for specific lipid types or components.

The environment is a volume which is the maximum number of vesicles from a single simulation (4096) times the size of the vesicle (usually 10000) divided by Avagadro's number divided by the total environmental lipid concentration, or usually 4.82×10^{-9} L.

3.3 Initial Vesicle Creation

Initial vesicles are seeded by selecting lipid molecules from the environment until the vesicle reaches a specific starting size. The vesicle starting size has gamma distribution with shape parameter 2 and a mean of the per-simulation specified starting size, with a minimum of 5 lipid molecules, or can be specified to have a precise number of molecules. Lipid molecules are then selected to be added to the lipid membrane according to four different methods. In the constant method, molecules are added in direct proportion to their concentration in the environment. The uniform method adds molecules in proportion to their concentration in the environment scaled by a uniform random value, whereas the random method adds molecules in proportion to a uniform random value. The final method is a

binomial method, which adjust the probability of adding a molecule of a specific component by the concentration of that component, and then adds the molecules one by one to the membrane. This final method is also used in the first three methods to add any missing molecules to the starting vesicle which were unallocated due to the requirement to add integer numbers of molecules. (For example, if a vesicle was to have 10 molecules split evenly between three lipid types, the 10th molecule would be assigned by randomly choosing between the three lipid types, weighted by their concentration in the environment.)

3.4 Simulation Step

Once the environment has been created and the initial vesicle has been formed, molecules join and leave the vesicle based on the kinetic parameters and state equation discussed above until the vesicle splits forming two progeny vesicles. The program then follows both vesicles and their progeny until the simulation reaches either the maximum number of vesicles (usually 4096), or the maximum simulation time (usually 100 s).

3.4.1 Calculation of Vesicle Properties

S_{vesicle} is the surface area of the vesicle, and is the sum of the surface area of all of the individual lipid components; each lipid type has a different surface area; we assume that the lipid packing is optimal, and there is no empty space.

For the other vesicle properties (length, unsaturation, charge, and curvature), we calculate the mean, the standard deviation, the mean of the log, the mean of the absolute value of the log, the standard deviation of the log, and the standard deviation of the absolute value of the log. All cases, when we take the log, we take the log of the absolute value, and map $\log(0)$ to 0. For the purpose of plotting vesicle properties, we only plot the mean of the property.

3.4.2 Joining and Leaving of Lipid Molecules

Determining the number of molecules to add to the lipid membrane (n_i) requires knowing $k_{\mathrm{f}i_{\text{adj}}}$, the surface area of the vesicle S_{vesicle} (see Section 3.4.1), the time interval dt during which lipids are added, the base $k_{\mathrm{f}i}$, and the concentration of the monomer in the environment $[C_{i\text{monomer}}]$ (see

Eq. (1)). $k_{\text{f}i\text{adj}}$ is calculated (see Eq. (2)) based on the vesicle properties and their hypothesized effect on the rate (in as many cases as possible, experimentally based) (see Section 2 for details). dt can be varied (see Section 3.4.3), but for a given step is constant. This leads to the following:

$n_i = k_{\text{f}i} k_{\text{f}i_{\text{adj}}} \left[C_{i_{\text{monomer}}} \right] S_{\text{vesicle}} \text{N}_{\text{A}} dt$

In the cases where $n_i > 1$, the integer number of molecules is added. Fractional n_i or the fractional remainder after the addition of the integer molecules are the probability of adding a specific molecule, and are compared to a uniformly distributed random value between 0 and 1. If the random value is less than or equal to the fractional part of n_i, an additional molecule is added.

Molecules leaving the vesicle are handled in a similar manner, with

$n_i = k_{\text{b}i} k_{\text{b}i_{\text{adj}}} C_{i_{\text{vesicle}}} \text{N}_{\text{A}} dt.$

Before addition or removal of molecules, the properties of the vesicle are calculated; this is done so that molecules can be considered to be added and removed at the same instant, even though additions are handled first programmatically. This also avoids cases where a removal would have resulted in a negative number of molecules for a particular type.

3.4.3 Step duration

dt is the time taken for each step of joining, leaving, and checking split. In the current implementation, it starts with a value of 10^{-6} s but this is modified in between each step if the number of molecules joining or leaving is too large or small. If more than half of the starting vesicle size molecules join or leave in a single step, dt is reduced by half. If less than the starting vesicle size molecules join or leave in 100 steps, dt is doubled. This is necessary to curtail run times and to automatically adjust to different experimental runs.

3.4.4 Vesicle splitting

If a vesicle has grown to a size which is more than double the starting vesicle size, the vesicle splits. Molecules are assigned to the progeny vesicles at random, with each progeny vesicle having an equal chance of getting a single molecule. The number of molecules to assign to the first vesicle has binomial distribution with a probability of an event in each trial of 0.5 and a number of trials equal to the number of molecules.

3.5 Output

The environment, initial vesicle, and the state of the vesicle immediately before and immediately after splitting are stored to produce later output.

Formalism

The most current revision of this formalism is available at `https://git.donarmstrong.com/ool/lipid_simulation_formalism.git`. This document is [git]• Branch: astrobiology_2017 @ cf5ae910 • Release: (2017-03-21).

References

1. Nichols, J. W. Thermodynamics and kinetics of phospholipid monomer-vesicle interaction. *Biochemistry* **24,** 6390–8 (Nov. 1985).
2. Estronca, L. M. B. B., Moreno, M. J. & Vaz, W. L. C. Kinetics and thermodynamics of the association of dehydroergosterol with lipid bilayer membranes. *Biophys J* **93,** 4244–53 (Dec. 2007).
3. Abreu, M. S. C., Moreno, M. J. & Vaz, W. L. C. Kinetics and thermodynamics of association of a phospholipid derivative with lipid bilayers in liquid-disordered and liquid-ordered phases. *Biophys J* **87,** 353–65 (July 2004).
4. Wimley, W. C. & Thompson, T. E. Exchange and flip-flop of dimyristoylphosphatidylcholine in liquid-crystalline, gel, and two-component, two-phase large unilamellar vesicles. *Biochemistry* **29,** 1296–303 (Feb. 1990).
5. Nichols, J. W. & Pagano, R. E. Use of resonance energy transfer to study the kinetics of amphiphile transfer between vesicles. *Biochemistry* **21,** 1720–6 (Apr. 1982).
6. Bai, J. & Pagano, R. E. Measurement of spontaneous transfer and transbilayer movement of BODIPY-labeled lipids in lipid vesicles. *Biochemistry* **36,** 8840–8 (July 1997).

7. Jones, J. D. & Thompson, T. E. Mechanism of spontaneous, concentration-dependent phospholipid transfer between bilayers. *Biochemistry* **29,** 1593–600 (Feb. 1990).

8. Smaby, J. M., Momsen, M. M., Brockman, H. L. & Brown, R. E. Phosphatidylcholine acyl unsaturation modulates the decrease in interfacial elasticity induced by cholesterol. *Biophys J* **73,** 1492–505 (Sept. 1997).

9. Cascales, J. J. L., Berendsen, H. J. C. & de la Torre, J. G. Molecular Dynamics Simulation of Water between Two Charged Layers of Dipalmitoylphosphatidylserine. *J. Phys. Chem.* **100,** 8621–7 (May 1996).

10. Pandit, S. A. & Berkowitz, M. L. Molecular dynamics simulation of dipalmitoylphosphatidylserine bilayer with Na+ counterions. *Biophys J* **82,** 1818–27 (Apr. 2002).

11. Demel, R. A., Paltauf, F. & Hauser, H. Monolayer characteristics and thermal behavior of natural and synthetic phosphatidylserines. *Biochemistry* **26,** 8659–65 (Dec. 1987).

12. Shaikh, S. R., Brzustowicz, M. R., Gustafson, N., Stillwell, W. & Wassall, S. R. Monounsaturated PE does not phase-separate from the lipid raft molecules sphingomyelin and cholesterol: role for polyunsaturation? *Biochemistry* **41,** 10593–602 (Aug. 2002).

13. Thurmond, R. L., Dodd, S. W. & Brown, M. F. Molecular areas of phospholipids as determined by 2H NMR spectroscopy. Comparison of phosphatidylethanolamines and phosphatidylcholines. *Biophys J* **59,** 108–13 (Jan. 1991).

14. Robinson, A. J., Richards, W. G., Thomas, P. J. & Hann, M. M. Behavior of cholesterol and its effect on head group and chain conformations in lipid bilayers: a molecular dynamics study. *Biophys J* **68,** 164–70 (Jan. 1995).

15. Thomas, P. D. & Poznansky, M. J. Cholesterol transfer between lipid vesicles. Effect of phospholipids and gangliosides. *Biochem J* **251,** 55–61 (Apr. 1988).

16. Huang, J. & Feigenson, G. W. A microscopic interaction model of maximum solubility of cholesterol in lipid bilayers. *Biophys. J* **76,** 2142–57 (Apr. 1999).

17. Huang, J., Buboltz, J. T. & Feigenson, G. W. Maximum solubility of cholesterol in phosphatidylcholine and phosphatidylethanolamine bilayers. *Biochim. Biophys. Acta* **1417,** 89–100 (Feb. 1999).

18. McConnell, H. & Radhakrishnan, A. Theory of the deuterium NMR of sterol-phospholipid membranes. *Proc Natl Acad Sci U S A* **103,** 1184–1189 (Jan. 2006).

19. McConnell, H. M. & Radhakrishnan, A. Condensed complexes of cholesterol and phospholipids. *Biochem. Biophys. Acta* **1610,** 159–73 (Mar. 2003).

20. Lund-Katz, S., Laboda, H. M., McLean, L. R. & Phillips, M. C. Influence of molecular packing and phospholipid type on rates of cholesterol exchange. *Biochemistry* **27,** 3416–23 (May 1988).

21. Israelachvili, J. N., Mitchell, D. J. & Ninham, B. W. Theory of Self-Assembly of Hydrocarbon Amphiphiles into Micelles and Bilayers. *J. Chem. Soc., Faraday Trans. 2,* **72,** 1525 (1976).

22. Kumar, V. V. Complementary molecular shapes and additivity of the packing parameter of lipids. *Proc Natl Acad Sci U S A* **88,** 444–8 (Jan. 1991).

23. Team, R. D. C. *R: A Language and Environment for Statistical Computing* ISBN 3-900051-07-0. R Foundation for Statistical Computing (Vienna, Austria, 2011). <`http://www.R-project.org`>.
